# Effect of Fertilization, Irrigation and Microbial Biostimulant on the Antioxidant Profile of Some Sweet Pepper Genotypes

**DOI:** 10.3390/plants15081278

**Published:** 2026-04-21

**Authors:** Marisa Jiménez-Pérez, Estela Moreno-Peris, Ana M. Adalid-Martínez, Ana Fita, María D. Raigón, Adrián Rodríguez-Burruezo

**Affiliations:** Instituto Universitario de Conservación y Mejora de la Agrodiversidad Valenciana (COMAV), Universitat Politècnica de València, Camino de Vera s/n, 46022 Valencia, Spain; majipe1@posgrado.upv.es (M.J.-P.); esmope1@upvnet.upv.es (E.M.-P.); anadmar@upv.es (A.M.A.-M.); anfifer@btc.upv.es (A.F.); mdraigon@qim.upv.es (M.D.R.)

**Keywords:** *Capsicum annuum*, antioxidant compounds, low fertilizer, irrigation reduction, PGPR biostimulant, ripening stage, traditional genotypes, sustainability

## Abstract

Sweet peppers (*Capsicum annuum* L.) are an important dietary source of antioxidants. Optimizing fruit antioxidant quality under reduced inputs is essential to valorize sustainable pepper production. Here, we evaluated seven Spanish genotypes (traditional/local, derived experimental hybrids and commercial hybrids) across six treatments combining two fertilization (100% and 50%) and irrigation (100% and 75%) regimes, with plant growth-promoting rhizobacteria (PGPR) applied under reduced fertilization treatments. Vitamin C and flavonoids were quantified by HPLC at the green-ripe and fully ripe stages, and carotenoids were determined spectrophotometrically at the fully ripe stage. Several genotypes largely maintained antioxidant content under stress treatments, whereas specific genotype × ripening stage combinations showed maximum increases in vitamin C (+102%), flavonoids (+86% for kaempferol) and carotenoids (+67% for yellow-orange carotenoids) under certain low-input treatments compared to the control. The PGPR effects on vitamin C and carotenoids were generally small, with occasional reductions. However, the PGPR increased total and some individual flavonoids by up to 96% (luteolin) in green-ripe Piquillo and 128% (quercetin) in fully ripe Isabel F1 fruits compared to the corresponding non-inoculated treatments. This multi-genotype, two ripening-stage evaluation identifies Spanish traditional germplasm and derived hybrids with stable or improved antioxidant profiles under low-input conditions and provides insight into PGPR effects. These results support the use of traditional genotypes in breeding for sustainable production.

## 1. Introduction

Sweet peppers (*Capsicum annuum* L.) are among the most widely consumed vegetables worldwide, valued for their particular flavor and diversity of colors and shapes [1]. Pepper is a horticultural crop of major economic importance worldwide. In 2024, the global pepper production exceeded 44.7 million tonnes, with an estimated production value of over USD 50.7 billion. Continental China was the leading producer, reaching around 17.3 million tonnes, while Spain ranked sixth, producing approximately 1.5 million tonnes [2]. They are also well-known sources of vitamin C, flavonoids and carotenoids (e.g., provitamin A carotenoids) [3,4,5], particularly at the fully ripe stage [6]. These compounds contribute to fruit nutritional quality and to plant physiology. Vitamin C comprises the reduced form, ascorbic acid, and its oxidized form, dehydroascorbic acid [7]. As a major water-soluble antioxidant, this redox couple contributes to cellular redox balance during plant development, fruit ripening and stress responses [8]. Flavonoids are a diverse group of polyphenolic secondary metabolites involved in plant growth, defense responses and seed development [9]. Carotenoids, lipophilic pigments ranging from yellow to red, are structural components of the photosynthetic apparatus in plants, contributing to light harvesting, photoprotection and the coloration of flowers and fruits [10].

These bioactive molecules are relevant to human health due to their antioxidant and other protective properties. Vitamin C is also important because of its roles in collagen synthesis, hormone production, gene transcription and iron absorption, among other functions. Its deficiency affects normal physiological functions and may ultimately lead to scurvy. Additionally, vitamin C has been associated with a protective role against certain cancers, cardiovascular diseases and infections [11]. Flavonoids can also induce different antioxidant enzymes and chelate metals such as iron or copper, reducing free radical formation. Accordingly, flavonoids have been associated with antioxidant, anti-carcinogenic and anti-inflammatory effects [12]. Regarding carotenoids, some of them can act as provitamin A precursors, contributing to dietary vitamin A intake. They have also been associated with protective effects against cardiovascular diseases, type 2 diabetes and certain cancers, as well as with positive effects on eye health [10,12]. Thus, a 100 g portion of fresh pepper can supply over 100% of the recommended daily intake of vitamin C [13] and make a meaningful contribution to provitamin A intake [14], according to the Food and Nutrition Board of National Academies [15]. However, fruit composition is strongly influenced by genotype, ripening stage and environmental conditions [16], making nutritional value dependent on both agronomic and genetic factors.

Intensive vegetable production has traditionally relied on high yields supported by high-input practices, including the extensive use of chemical fertilizers and irrigation water, which has resulted in multiple environmental risks [17]. Ensuring food production for a growing population while reducing the use of natural resources has therefore become a major challenge for the agricultural sector [18]. This concern is particularly critical in countries such as Spain, which faces strong spatial and temporal variability in water availability, with recurrent droughts severely affecting southeastern regions such as Almería, Murcia and Alicante [19]. These pressures also affect greenhouse horticultural production, a key component of agriculture in southeastern Spain, as in other Mediterranean countries. Moreover, greenhouses only partially control growing environment and still depend on external climatic conditions [20]. Consequently, adaptation strategies are necessary to avoid detrimental impacts on yield, fruit nutritional quality and postharvest performance [20,21]. Many of these strategies aim to improve resource use efficiency, including fertilization and irrigation optimization [20]. Overall, the combined challenges of intensive farming and global warming in these areas are driving the urgent development of more sustainable and resilient agroecosystems [22].

Alongside the transition to lower input farming systems, the development of crop varieties better adapted to limited resources and with improved resistance or tolerance to pests and diseases is essential. Such advances would reduce dependence on agricultural inputs, such as chemical fertilizers and pesticides [20,23]. Screening and breeding pepper genotypes better adapted to sustainable farming is particularly important, as pepper yield is highly sensitive to multiple factors, including nutrient and water deficit [24]. Thus, traditional cultivars and landraces are valuable reservoirs of genetic variation for traits suited to current climatic instability [25], and they are also associated with distinctive fruit nutritional quality profiles [26].

Spain, as a secondary center of diversity for *Capsicum annuum*, has numerous ecotypes adapted to diverse agronomic and climatic conditions. Some of them are highly appreciated by consumers and hold Protected Geographical Indications (PGIs) and Protected Designations of Origin (PDOs), such as Padrón-Herbón (Galicia), Bola (Murcia), Piquillo de Lodosa (Navarra), Riojano (La Rioja), among others [27]. However, many of these genotypes remain underrepresented in studies evaluating fruit nutritional quality at the two main commercial ripening stages under realistic, moderately low-input conditions, particularly in greenhouse systems of southeastern Spain.

In addition to advancements in breeding tools, plant biostimulants have emerged as a promising sustainable strategy to improve crop yield and quality while reducing environmental impact [28]. They are defined as microorganisms or substances that enhance plant growth without including pesticides, nutrients and soil improvers [29]. Among them, microbial biostimulants, particularly plant growth-promoting rhizobacteria (PGPR), are of particular interest due to their natural associations with plant roots [30]. PGPR effects are associated with several mechanisms, including those related to nutrient acquisition, such as phosphorus and other micronutrient solubilization, atmospheric nitrogen fixation and siderophore production, which contribute to the stabilization and plant availability of Fe^3+^ ions. They are also associated with the production or modulation of phytohormones such as auxins, cytokinins, abscisic acid (ABA) or ethylene. These compounds can influence root system development and the uptake of nutrients and water [31]. In addition, PGPR can induce plant defense responses, such as the induced systemic resistance (ISR), with potential effects on redox regulation and secondary metabolism [32]. PGPR efficacy depends on the strain, environmental conditions and host genotype, as well as on successful rhizosphere colonization and compatibility with the native soil microbiome [31,33]. All these factors, together with the complexity of antioxidant regulation, could explain the variable and sometimes contradictory PGPR effects reported for these compounds [34,35]. Moreover, in sweet pepper, evidence on PGPR effects on fruit antioxidant composition remains limited, particularly under low-input greenhouse conditions across diverse Spanish germplasm and considering ripening stages. In this context, we used a commercial PGPR consortium including genera widely applied as microbial biofertilizers to improve nutrient availability and uptake (i.e., *Pseudomonas*, *Bacillus* and *Azospirillum*) [36], which have also been reported to mitigate water deficit effects [32].

Therefore, complementary to our previous work in this trial [37], which reported agronomic performance, rhizosphere enzymatic activities and fruit sugar profiles of this collection, we focused here on fruit antioxidant quality. In this study, we aimed to identify Spanish sweet pepper genotypes with robust antioxidant profiles under agronomically realistic, moderate input reductions in fertilization (100% and 50%) and irrigation (100% and 75%), selected as feasible greenhouse input-saving scenarios. Additionally, we evaluated whether PGPR inoculation could further support fruit antioxidant quality under low-input management. Given the intended use of these microbial strains as biofertilizers under stress, and to keep the experimental design manageable, inoculation was implemented only in the 50% fertilization treatments. Thus, PGPR responses were interpreted within reduced fertilization conditions and were not extrapolated to full fertilization. We quantified vitamin C and key flavonoids at the green-ripe and fully ripe (red) stages, the two main commercial ripening stages, and carotenoids at the fully ripe stage, in a collection comprising Spanish traditional/local genotypes, derived experimental hybrids and commercial hybrids. This study extends the characterization of this collection under low-input management and PGPR application. It provides a basis for identifying the genotypes that best maintain fruit internal quality (i.e., fruit antioxidant composition) and for further exploring Spanish traditional/local germplasm as a source of alleles for breeding programs targeting sustainable production systems.

## 2. Results

### 2.1. Effects of Genotype, Treatment, Ripening Stage and Their Interactions on the Studied Traits

On the whole, the general three-way ANOVA showed significant effects of all the main factors: genotype (G), treatment (T) and ripening stage (R) on the evaluated bioactive compounds (Table 1). According to the mean squares (MS), the ripening stage was generally the main contributor to the variation of most antioxidants, followed by the genotype.

Exceptions were observed for luteolin and total flavonoids, for which the genotype was the main contributor. Finally, the treatment also contributed considerably to variation in vitamin C, quercetin and kaempferol (Table 1).

Among interactions, the T × R interaction had a strong effect on vitamin C, quercetin and kaempferol, indicating specific accumulation patterns depending on treatment and ripening stage. In addition, both the G × T and, especially, the G × R interactions significantly contributed to the variation in vitamin C (Table 1), reflecting genotype-dependent accumulation patterns across treatments and ripening stages. By contrast, the G × T effects were significant only for quercetin, kaempferol and apigenin, while G × R showed variable effects among flavonoids, with quercetin being the only flavonoid not significantly affected (Table 1).

Given the strong impact of the ripening stage on most antioxidants, separate ANOVAs were also performed for each ripening stage to minimize biases in estimating the effects of genotype and treatment (Table 1). Carotenoids were analyzed only at the fully ripe stage, when their accumulation is predominant.

At the green-ripe stage, the results were consistent with the general ANOVA, showing significant effects of genotype and treatment for all compounds. Genotype generally contributed the most to variation, followed by treatment and, to a lesser extent, by the G × T interaction. The significant G × T interaction for vitamin C indicated specific genotype responses to treatments. For flavonoids, G × T effects were generally weak, as they were not significant for luteolin and kaempferol, whereas quercetin, apigenin and total flavonoids showed significant, but smaller, G × T terms (Table 1).

By contrast, at the fully ripe stage, the treatment was the main contributor to the variation in vitamin C, quercetin and kaempferol, while the genotype was the most important one to luteolin, apigenin, total flavonoids and red, yellow-orange and total carotenoids. The G × T interaction significantly influenced only some compounds, mainly vitamin C and carotenoids, while showing a weaker effect for flavonoids, with no significant effects on quercetin, luteolin and total flavonoids (Table 1).

### 2.2. Vitamin C Content

**Figure 1:**
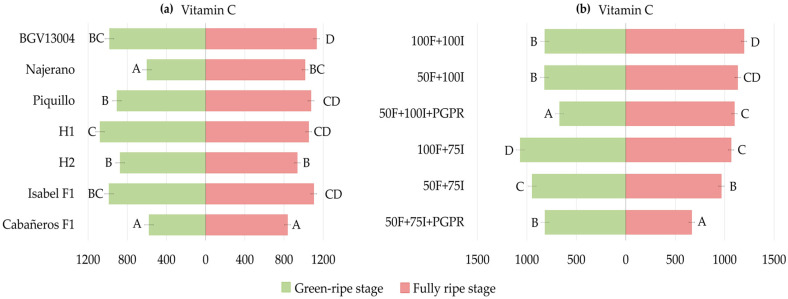
(**a**) Genotype means across treatments for vitamin C (mg kg^−1^) at the green-ripe (left; green) and fully ripe (right; red) stages (LS means ± LS SE, *n* = 18). (**b**) Treatment means across genotypes for vitamin C (mg kg^−1^) at the green-ripe (left; green) and fully ripe (right; red) stages (LS means ± LS SE, *n* = 21). Different capital letters indicate significant differences among genotypes within each stage in (**a**) and among treatments within each stage in (**b**) (Duncan’s test, *p* < 0.05).

The considerable variability observed in vitamin C content among genotypes at both ripening stages was supported by the significant genotype effect detected in the ANOVA. At the green-ripe stage, genotype means across treatments ranged from 578 and 600 mg kg^−1^ in Cabañeros F1 and Najerano, respectively, to 1078 mg kg^−1^ in H1. At the fully ripe stage, vitamin C increased in all genotypes. Cabañeros F1 again showed the lowest mean value (836 mg kg^−1^) and BGV13004 the highest (1132 mg kg^−1^) (Figure 1a, Table S1).

Regarding treatment effects, vitamin C differed significantly at both ripening stages, in agreement with the ANOVA. At the green-ripe stage, vitamin C averaged 819 mg kg^−1^ across genotypes in the control (100F + 100I, reference baseline). Relative to the control, vitamin C increased by 30% under low irrigation (100F + 75I; 1068 mg kg^−1^) and by 16% under combined fertilizer and irrigation reduction (50F + 75I; 948 mg kg^−1^). By contrast, values under fertilizer reduction (50F + 100I) remained comparable to 100F + 100I. Under 50F + 100I + PGPR, vitamin C reached 670 mg kg^−1^, corresponding to an approximately 20% reduction compared to 50F + 100I and the control. Under 50F + 75I + PGPR, it averaged 818 mg kg^−1^, 14% lower than under 50F + 75I (Figure 1b, Table S2).

At the fully ripe stage, vitamin C reached 1196 mg kg^−1^ in the control and decreased under 100F + 75I and 50F + 75I to 1066 and 967 mg kg^−1^, respectively (11% and 19% below the control). Again, 50F + 100I showed contents comparable to the control. Under 50F + 100I + PGPR, vitamin C averaged 1099 mg kg^−1^, similar to 50F + 100I but slightly lower than the control. It also decreased to 667 mg kg^−1^ under 50F + 75I + PGPR, 31% and 44% lower than under 50F + 75I and 100F + 100I, respectively (Figure 1b, Table S2).

Genotype-specific responses were also observed, although most genotypes remained stable in comparison to the control within each stage. At the green-ripe stage, 50F + 100I increased vitamin C only in BGV13004 and H1, by 75% and 48%, respectively, compared to the control. The strongest positive responses were observed under 100F + 75I, with increases of 75–102% in BGV13004, Najerano and H1 compared to 100F + 100I. Under 50F + 75I, only H1 increased vitamin C, by 74% (Table 2 and Table S3). At the fully ripe stage, most genotypes remained stable under 50F + 100I, except for BGV13004 and Cabañeros F1, which decreased by approximately 27% compared to the control. Under 100F + 75I, Isabel F1 increased vitamin C by 24% compared to 100F + 100I, whereas BGV13004, Piquillo and Cabañeros F1 decreased it by 17–41%. Under 50F + 75I, reductions were observed only in BGV13004 and H2, both around 40% below 100F + 100I (Table 3 and Table S4).

Overall, PGPR inoculation within reduced fertilization treatments showed limited and genotype-dependent variations in vitamin C. In green-ripe fruits, a significant decrease was only detected in BGV13004 under 50F + 100I + PGPR compared to 50F + 100I (−54%), although values remained comparable to 100F + 100I (Table 2 and Table S3). At the fully ripe stage, PGPR treatments showed more variation across treatments and genotypes. Thus, in Cabañeros F1, vitamin C was 23% higher under 50F + 100I + PGPR than under 50F + 100I, but 13% lower than in the control. By contrast, 50F + 75I + PGPR reduced vitamin C in several genotypes compared to 50F + 75I, including H1, Isabel F1 and Cabañeros F1 (36%, 61% and 64% lower than under 50F + 75I, respectively). These values were also 46%, 63% and 68% lower than under 100F + 100I, respectively (Table 3 and Table S4).

### 2.3. Main Flavonoids (Quercetin, Luteolin, Kaempferol and Apigenin)

**Figure 2:**
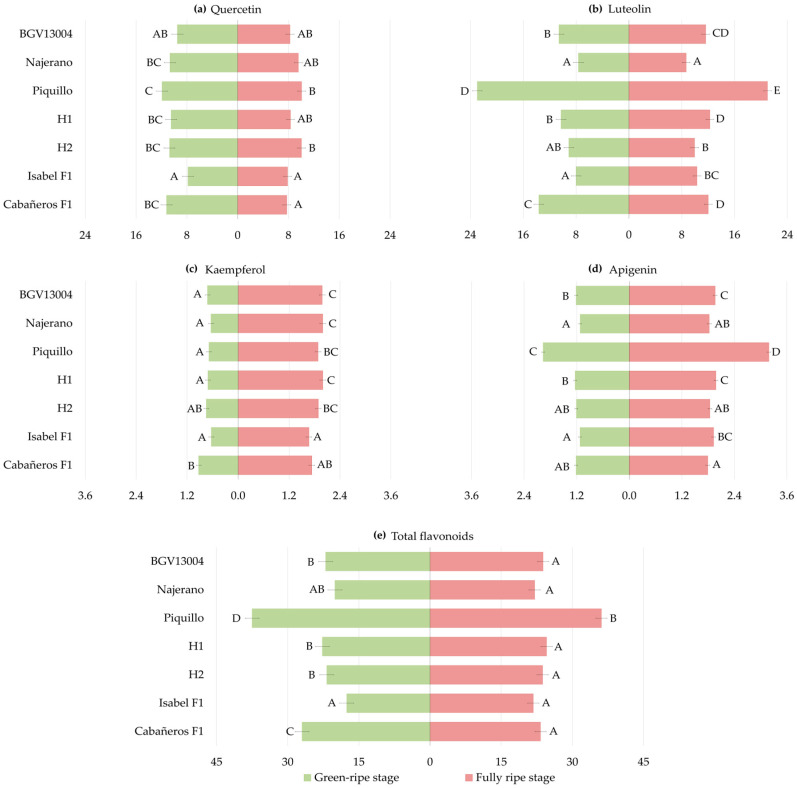
Genotype means across treatments for (**a**) quercetin, (**b**) luteolin, (**c**) kaempferol, (**d**) apigenin and (**e**) total flavonoids (mg kg^−1^) at the green-ripe (left; green) and fully ripe (right; red) stages (LS means ± LS SE, *n* = 18). Different capital letters indicate significant differences among genotypes within each stage (Duncan’s test, *p* < 0.05).

Genotype means were consistent with the significant genotype effects detected in the ANOVA. At the green-ripe stage, quercetin means across treatments ranged from 7.83 mg kg^−1^ in Isabel F1 to 11.92 mg kg^−1^ in Piquillo (Figure 2a, Table S1). Luteolin ranged from 7.64–7.98 mg kg^−1^ in Najerano and Isabel F1 to 22.95 mg kg^−1^ in Piquillo (Figure 2b, Table S1). Kaempferol was relatively uniform among genotypes, ranging from 0.63–0.72 mg kg^−1^ in Isabel F1, Najerano, Piquillo, H1 and BGV13004 to 0.93 mg kg^−1^ in Cabañeros F1 (Figure 2c, Table S1). Apigenin varied from 1.12 mg kg^−1^ in Najerano and Isabel F1 to 1.97 mg kg^−1^ in Piquillo (Figure 2d, Table S1), whereas total flavonoids were lowest in Isabel F1 (17.57 mg kg^−1^) and highest in Piquillo (37.52 mg kg^−1^) (Figure 2e, Table S1).

At the fully ripe stage, patterns varied across compounds. Quercetin ranged from 7.73–7.86 mg kg^−1^ in Cabañeros F1 and Isabel F1 to 10.04–10.06 mg kg^−1^ in H2 and Piquillo, respectively (Figure 2a, Table S1). Luteolin ranged from 8.68 mg kg^−1^ in Najerano to 20.98 mg kg^−1^ in Piquillo (Figure 2b, Table S1). Kaempferol varied from 1.67 mg kg^−1^ in Isabel F1 to 1.98–2.00 mg kg^−1^ in BGV13004, Najerano and H1 (Figure 2c, Table S1), while apigenin was lowest in Cabañeros F1 (1.79 mg kg^−1^), and highest in Piquillo (3.19 mg kg^−1^) (Figure 2d, Table S1). Finally, total flavonoids were highest in Piquillo (36.12 mg kg^−1^), whereas the remaining genotypes accumulated 21.76–24.56 mg kg^−1^ (Figure 2e, Table S1).

**Figure 3:**
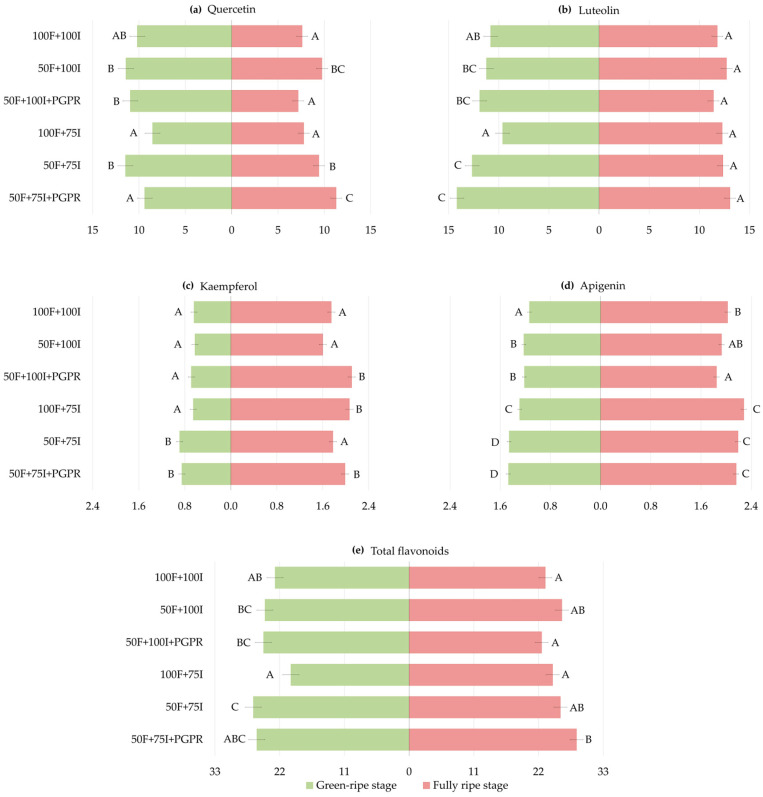
Treatment means across genotypes for (**a**) quercetin, (**b**) luteolin, (**c**) kaempferol, (**d**) apigenin and (**e**) total flavonoids (mg kg^−1^) at the green-ripe (left; green) and fully ripe (right; red) stages (LS means ± LS SE, *n* = 21). Different capital letters indicate significant differences among treatments within each stage (Duncan’s test, *p* < 0.05).

A general treatment effect was observed for most flavonoids at both ripening stages, corroborating the ANOVA. At the green-ripe stage, quercetin averaged 10.20 mg kg^−1^ across genotypes in the control and remained broadly stable across stress treatments. Under 50F + 75I + PGPR (9.40 mg kg^−1^), quercetin was 18% lower than under 50F + 75I (Figure 3a, Table S2). Luteolin averaged 10.82 mg kg^−1^ in the control and increased by 17% under 50F + 75I (12.66 mg kg^−1^). Under 50F + 75I + PGPR (14.18 mg kg^−1^), luteolin was 31% higher than under 100F + 100I (Figure 3b, Table S2). Similarly, kaempferol was 0.64 mg kg^−1^ in the control, and it increased by 39% under 50F + 75I (0.89 mg kg^−1^). Under 50F + 75I + PGPR, it reached 0.85 mg kg^−1^, 33% higher than in the control (Figure 3c, Table S2). Apigenin was lowest under 100F + 100I (1.13 mg kg^−1^) and increased slightly under 50F + 100I and 100F + 75I, reaching 1.46 mg kg^−1^ under 50F + 75I (29% higher than the control). Under PGPR inoculation, it reached 1.47 mg kg^−1^ in 50F + 75I + PGPR, corresponding to a 30% increase compared to 100F + 100I (Figure 3d, Table S2). Finally, total flavonoids averaged 22.79 mg kg^−1^ under 100F + 100I. Only a significant increase of 16% under 50F + 75I (26.48 mg kg^−1^) was observed. PGPR inoculation did not significantly modify total flavonoids compared to the corresponding non-inoculated stress treatments (Figure 3e, Table S2).

At the fully ripe stage, quercetin averaged 7.60 mg kg^−1^ in the control, increasing by about 26% under 50F + 100I (9.74 mg kg^−1^) and 50F + 75I (9.41 mg kg^−1^). Under PGPR treatments, quercetin decreased to 7.18 mg kg^−1^ under 50F + 100I + PGPR, 26% lower than under 50F + 100I. However, it reached 11.27 mg kg^−1^ under 50F + 75I + PGPR, 20% and 48% higher than under 50F + 75I and the control, respectively (Figure 3a, Table S2). By contrast, luteolin did not differ among treatments (Figure 3b, Table S2). Kaempferol averaged 1.75 mg kg^−1^ in the control, increasing by 18% under 100F + 75I (2.06 mg kg^−1^). Under 50F + 100I + PGPR, it reached 2.10 mg kg^−1^, which was 31% and 20% higher than 50F + 100I (1.60 mg kg^−1^) and 100F + 100I, respectively. Under 50F + 75I + PGPR, it increased to 1.99 mg kg^−1^, about 13% higher than 50F + 75I (1.77 mg kg^−1^) and 100F + 100I (Figure 3c, Table S2). Apigenin averaged 2.03 mg kg^−1^ under 100F + 100I and increased slightly under 100F + 75I and 50F + 75I (2.28 and 2.19 mg kg^−1^, respectively). Under PGPR treatments, apigenin was 1.85 mg kg^−1^ under 50F + 100I + PGPR and 2.16 mg kg^−1^ under 50F + 75I + PGPR, indicating a slight decrease and increase compared to 100F + 100I, respectively (Figure 3d, Table S2). Total flavonoids did not differ among treatments, except under 50F + 75I + PGPR, where they reached 28.47 mg kg^−1^, 23% higher than in the control (Figure 3e, Table S2).

Genotype-specific responses were observed across compounds and ripening stages. At the green-ripe stage, responses under 50F + 100I compared to 100F + 100I were limited, with increased apigenin in H1 by 25%, and higher quercetin, kaempferol and total flavonoids in H2 (50–82%). Under 100F + 75I, quercetin, luteolin and total flavonoids were largely unchanged. However, kaempferol increased in H2 by 64% but decreased in Piquillo by 25%, compared to 100F + 100I. Apigenin also increased in Najerano, H1 and Cabañeros F1 by 26–38%. Under 50F + 75I, luteolin increased only in H2, by 68% compared to 100F + 100I. Kaempferol rose in H1 and H2 by 78% and 86%, respectively. Apigenin increased in BGV13004, Najerano, Piquillo, H1 and H2 by 17–62%, while total flavonoids rose only in H2 (49%) (Table 4 and Table S5).

At the fully ripe stage, 50F + 100I increased quercetin only in H2 by 78% compared to the control, whereas Najerano showed a 11% decrease in apigenin. Under 100F + 75I, kaempferol rose in BGV13004, Najerano and Isabel F1 by 36–68% compared to 100F + 100I, while apigenin increased by 16–30% in BGV13004, H1 and H2. By contrast, quercetin decreased in Piquillo by 43%. Under 50F + 75I, luteolin increased only in Cabañeros F1 by 49% compared to the control, whereas kaempferol increased in Isabel F1 and Cabañeros F1 by 32% and 53%, respectively. Apigenin rose by around 27% in H1 and Cabañeros F1, while total flavonoids remained unchanged (Table 5 and Table S6).

Genotype-specific variations were observed under PGPR inoculation under reduced fertilization treatments. At the green-ripe stage, the strongest responses under 50F + 75I + PGPR were observed in Piquillo and H2. In Piquillo, quercetin, luteolin, apigenin and total flavonoids significantly increased compared to 50F + 75I and/or 100F + 100I. The largest increases were observed for luteolin, which was 96% and 89% higher, respectively, than under 50F + 75I and the control, while total flavonoids increased by 77% and 69%, respectively. By contrast, H2 showed marked decreases in quercetin, kaempferol and total flavonoids, including reductions of 54% and 40% in quercetin compared to 50F + 75I and 100F + 100I, respectively, and a 48% decrease in total flavonoids compared to 50F + 75I. Najerano also showed a 40% decrease in quercetin compared to 50F + 75I (Table 4 and Table S5).

At the fully ripe stage, quercetin showed contrasting responses, decreasing in Piquillo under 50F + 100I + PGPR, by about 46% compared to both 50F + 100I and 100F + 100I, and by 43% in H2 compared to 50F + 100I. Under 50F + 75I + PGPR, quercetin increased in Isabel F1, being 128% and 71% higher than under 50F + 75I and the control, respectively. By contrast, luteolin was largely unchanged. Kaempferol increased in several genotypes under PGPR treatments. Thus, under 50F + 100I + PGPR, Najerano, Isabel F1 and Cabañeros F1 increased kaempferol by 46–74% compared to 50F + 100I and by 49–73% compared to 100F + 100I. Under 50F + 75I + PGPR, kaempferol increased in Najerano and Isabel F1, being 41% and 23% higher than under 50F + 75I, and 47% and 63% higher than in the control, respectively. Apigenin did not show significant differences under PGPR compared to the corresponding non-inoculated treatments, while for total flavonoids, Piquillo decreased by about 35% under 50F + 100I + PGPR compared to 50F + 100I and 100F + 100I, while their content rose in Isabel F1 by 48% and 34% under 50F + 75I + PGPR in relation to 50F + 75I and 100F + 100I, respectively (Table 5 and Table S6).

Overall, these genotype-specific patterns supported the significant G × T interactions detected in the ANOVAs (Table 1, Table 4 and Table 5).

### 2.4. Total Carotenoids (Red and Yellow-Orange Carotenoids)

**Figure 4:**
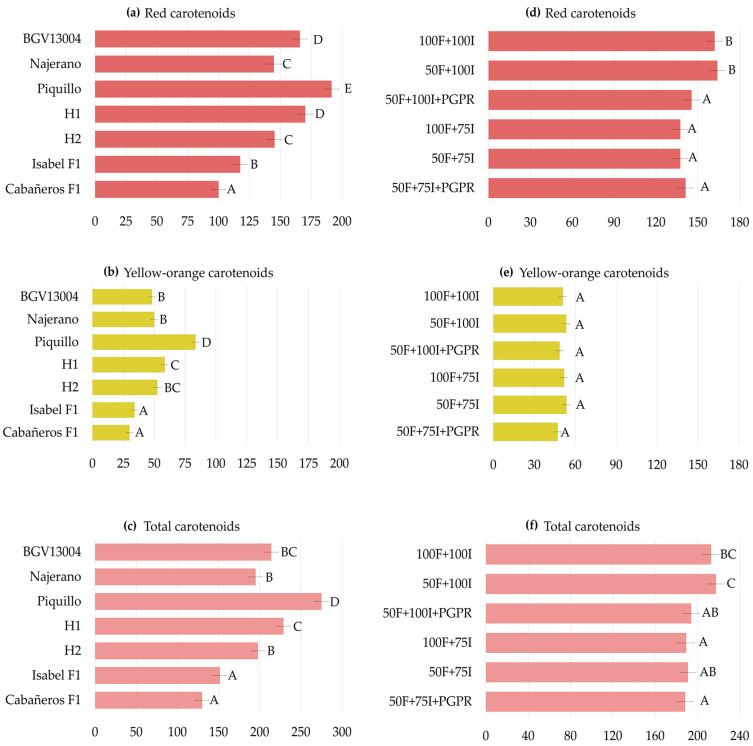
Genotype means across treatments for (**a**) red, (**b**) yellow-orange and (**c**) total carotenoids (mg kg^−1^) at the fully ripe stage (LS means ± LS SE, *n* = 18). Treatment means across genotypes for (**d**) red, (**e**) yellow-orange and (**f**) total carotenoids at the fully ripe stage (LS means ± LS SE, *n* = 21). Different capital letters indicate significant differences among genotypes (**a**–**c**) and among treatments (**d**–**f**) (Duncan’s test, *p* < 0.05).

In agreement with the ANOVA, the genotype factor had a significant influence on carotenoid profiles. Averaged across treatments, Cabañeros F1 showed the lowest red carotenoid content (100 mg kg^−1^), whereas Piquillo reached the highest value (191 mg kg^−1^) (Figure 4a and Table S1). Yellow-orange carotenoids followed the same pattern, ranging from 30–34 mg kg^−1^ in Cabañeros F1 and Isabel F1 to 83 mg kg^−1^ in Piquillo (Figure 4b and Table S1). Similarly, total carotenoids varied from 130–151 mg kg^−1^ in Cabañeros F1 and Isabel F1 to 275 mg kg^−1^ in Piquillo (Figure 4c and Table S1).

Across treatments, red and total carotenoids also varied significantly. Under control conditions, red carotenoids averaged 162 mg kg^−1^. Compared to 100F + 100I, they decreased by 15% under both 100F + 75I and 50F + 75I (137 mg kg^−1^) and remained comparable under 50F + 100I. Under PGPR inoculation, red carotenoids averaged 145 mg kg^−1^ under 50F + 100I + PGPR, about 12% lower than under 50F + 100I and the control. Under 50F + 75I + PGPR, they reached 141 mg kg^−1^, being 13% lower than under 100F + 100I (Figure 4d and Table S2). Yellow-orange carotenoids were 51 mg kg^−1^ in the control and ranged from 47 to 54 mg kg^−1^ across treatments, with no significant differences (Figure 4e and Table S2). Total carotenoids averaged 213 mg kg^−1^ in the control and showed only minor changes across treatments, highlighting a 11% decrease under 100F + 75I (189 mg kg^−1^). Under PGPR inoculation, total carotenoids decreased by 11% under 50F + 100I + PGPR (194 mg kg^−1^) compared to 50F + 100I (217 mg kg^−1^) and 12% under 50F + 75I + PGPR (188 mg kg^−1^) in comparison to the control (Figure 4f and Table S2).

Genotype-specific responses were also observed. Under 50F + 100I, few differences were detected compared to 100F + 100I, except for H1, which increased both red and total carotenoids by 43%. Under 100F + 75I, BGV13004 reduced red, yellow-orange and total carotenoids by approximately 45% compared to the control. Similarly, Piquillo reduced red and total carotenoids by 24%. By contrast, Najerano increased yellow-orange carotenoids by 67%. Under 50F + 75I, few changes were detected, except for BGV13004, in which red and total carotenoids decreased by around 27% compared to 100F + 100I (Table 6 and Table S7).

PGPR inoculation generally showed stable or lower carotenoid levels compared to their non-inoculated stress treatments. In H1, red carotenoids were 26% lower under 50F + 100I + PGPR than under 50F + 100I. Piquillo also showed a 31% reduction under 50F + 100I + PGPR compared to 50F + 100I and 100F + 100I. Yellow-orange carotenoids also decreased in Piquillo under 50F + 100I + PGPR, by 31% compared to 50F + 100I. Total carotenoids followed similar patterns. In Piquillo, they were about 30% lower under 50F + 100I + PGPR than under 50F + 100I and 100F + 100I, while in H1, total carotenoids decreased by 24% compared to 50F + 100I (Table 6 and Table S7).

The variable genotype-specific responses were in line with the significant G × T interactions detected by the ANOVA in most compounds, reflecting the differential impact of treatments on carotenoid accumulation across the collection (Table 1 and Table 6).

### 2.5. Spearman’s Correlations

**Figure 5:**
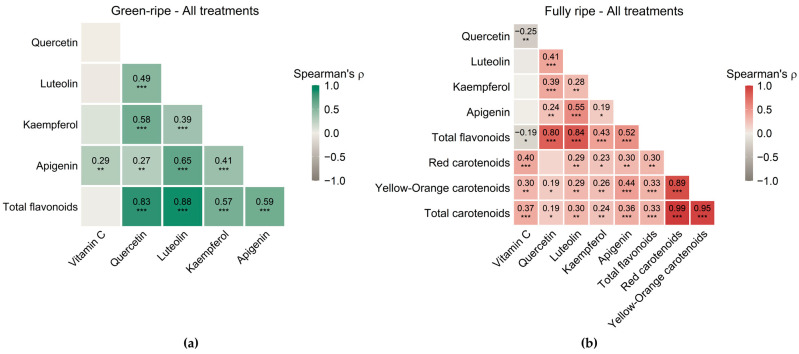
Spearman’s rank correlation (*ρ*) heatmap for (**a**) vitamin C and flavonoids at the green-ripe stage and (**b**) vitamin C, flavonoids and carotenoids at the fully ripe stage. *, ** and *** indicate FDR-adjusted *p* < 0.05, 0.01 and 0.001, respectively.

Correlations among the evaluated antioxidants differed between ripening stages and treatment categories (Figure 5 and Figure S1). At the green-ripe stage, vitamin C showed no relevant correlations with flavonoids, except for a modest association with apigenin (*ρ* = 0.29). However, flavonoids were strongly intercorrelated, and total flavonoids correlated most strongly with quercetin and luteolin. The highest individual flavonoids correlations were observed between luteolin and apigenin (*ρ* = 0.65), quercetin and kaempferol (*ρ* = 0.58), followed by quercetin and luteolin (*ρ* = 0.49) (Figure 5a). When data were analyzed separately by treatment category (control: 100F + 100I; low-input without PGPR: 50F + 100I, 100F + 75I, 50F + 75I; reduced fertilization with PGPR: 50F + 100I + PGPR and 50F + 75I + PGPR), the correlation between vitamin C and apigenin remained significant only in the Low-input without PGPR category (*ρ* = 0.29), whereas the main flavonoid associations (quercetin and kaempferol, luteolin and apigenin, or quercetin and luteolin) were consistently observed across categories (Figure S1a–c).

At the fully ripe stage, vitamin C showed a negative association with quercetin (*ρ* = −0.25) and total flavonoids (*ρ* = −0.19), whereas it correlated positively with red, yellow-orange and total carotenoids (*ρ* = 0.30–0.40). Flavonoids remained intercorrelated, and total flavonoids correlated most again with quercetin and luteolin. Among individual flavonoids, luteolin and apigenin showed a strong association (*ρ* = 0.55), and moderate correlations were observed between quercetin and luteolin (*ρ* = 0.41) and between quercetin and kaempferol (*ρ* = 0.39). Regarding flavonoid and carotenoid relationships, apigenin showed the highest correlations (*ρ* = 0.30–0.44), followed by total flavonoids (*ρ* = 0.30–0.33), luteolin (*ρ* = 0.29–0.30) and kaempferol (0.23–0.26). As expected, red and yellow-orange carotenoids were strongly correlated with each other and with total carotenoids (Figure 5b). When analyses were stratified by treatment category, a distinct negative association between quercetin and vitamin C emerged in reduced fertilization with PGPR (*ρ* = −0.49). By contrast, positive correlations between vitamin C and carotenoids were mainly observed in the low-input without PGPR category. Similarly, some intercorrelations among individual flavonoids were more pronounced in low-input without PGPR. Correlations between flavonoids and carotenoids were particularly evident for apigenin in low-input without PGPR (*ρ* = 0.37–0.47), whereas kaempferol showed higher correlations with carotenoids mainly in the control (*ρ* = 0.68–0.70). Finally, correlations among carotenoids remained strong across treatment categories (Figure S1d–f).

## 3. Discussion

Agroecosystems are highly vulnerable to climate change [38], due to the rising temperatures, water salinization, increasingly erratic drought and rainfall patterns [39] and soil degradation [40]. This vulnerability also extends to partially protected cultivation systems, including Mediterranean greenhouses [20]. More sustainable management is needed to ensure long-term productivity and ecosystem resilience, in line with the United Nations 2030 Agenda for Sustainable Development [41]. Achieving these goals requires a holistic approach that combines improved greenhouse environmental control [20], the conservation of genetic diversity, the use and breeding of resilient genotypes, optimized resource use and innovative agronomic strategies [42], including plant biostimulants [43]. Sustainable crop management aims not only to maintain yield while optimizing inputs such as fertilizer and irrigation [44], but also to preserve or enhance fruit quality, including internal nutritional quality and postharvest traits (e.g., physiological disorders and shelf-life performance) that affect market value and consumer acceptance [21,45]. Moreover, fruit composition, firmness and storability can be strongly affected by preharvest conditions, such as the irrigation, nutrient balance, temperature, light intensity and humidity [21]. Here, we focus on fruit internal quality, specifically the antioxidant profile.

Given the distinct genetic backgrounds represented in our collection, which included Spanish traditional/local cultivars previously characterized for genetic diversity and varietal typification [27,46], together with experimental and commercial hybrids, genotype-dependent responses across treatments were expected. These patterns may reflect differences in constitutive antioxidant status and in the developmental regulation of ascorbate (vitamin C), phenylpropanoid/flavonoid and carotenoid metabolisms [47,48,49]. Mechanistically, these compounds play complementary roles within a coordinated antioxidant network involved in ROS control and redox homeostasis. Ascorbate also interacts with different phytohormone pathways. Flavonoids, particularly those with a dihydroxy B-ring, such as quercetin and luteolin, can limit lipid oxidative damage. Carotenoids also contribute to photoprotection and further reduce lipid peroxidation [50]. Responses under the tested low-input conditions may be influenced by differential sensitivity and responsiveness among genotypes to nutrient and water limitation [51] and by PGPR signals that can modulate pathways related to stress and defense [52].

A general tendency to accumulate vitamin C and phenolic compounds under nitrogen limitation has been widely reported. Nitrogen deprivation can induce oxidative stress and antioxidant responses, including shifts in ascorbate metabolism and recycling (e.g., the ascorbate-glutathione cycle) [7]. In pepper fruits, nitrogen limitation has been linked to higher abundance of ascorbic acid precursors, and responses appear to be ripening-dependent [53]. In parallel, low nitrogen often promotes the accumulation of carbon-based phenolics/flavonoids, sometimes interpreted as a photoprotective response [54]. However, fertilizer effects on carotenoids are less consistent. This may be because carotenoid accumulation is strongly dependent on ripening and varies with stress intensity and genotype. But, enhanced carotenoid biosynthesis under low nitrogen has also been described [55,56]. In our study, some genotypes showed significant increases in vitamin C, as also reported in tomato by Flores et al. [57], but also reductions under stress conditions, particularly at the fully ripe stage, as previously observed in mature pepper fruits [58]. Similarly, some flavonoids increased under nutrient limitation at the green-ripe stage, consistent with observations in early tomato development [59]. However, most genotypes maintained stable concentrations of vitamin C, flavonoids and carotenoids under reduced fertilization, with only specific variations. This stability agreed with previous findings in pepper [60], and other crops [61,62].

Under reduced irrigation, accumulation of vitamin C and flavonoids has frequently been reported, often linked to ROS scavenging [63,64]. For carotenoids, some studies have described detrimental effects [65], whereas others have reported increases under oxidative conditions [66]. Carotenoids act as precursors of abscisic acid (ABA), which typically accumulates under drought. Moreover, ABA signaling can modulate the expression of carotenoid pathway genes. Thus, the variability observed in carotenoids may partly reflect their roles in both pigmentation and drought responses [67]. In our work, only a subset of genotypes showed significant changes. By contrast, several compounds remained stable in part of the sweet pepper collection across treatments and/or ripening stages, particularly for quercetin, luteolin and carotenoids. Similar stability in vitamin C was reported by Kopta et al. [68] and Agyemang Duah et al. [69] in most pepper and chili cultivars under deficit irrigation. Medina-Lozano et al. [70] also found that drought reduced vitamin C in some lettuce varieties, while others maintained stable contents. In our collection, vitamin C increases were more evident at the green-ripe stage, whereas responses at full ripening were smaller and occasionally negative. A stronger negative effect of water deficit at advanced ripening has also been reported in pepper [71]. This stage dependence could reflect the developmental regulation of ascorbate metabolism during pepper fruit ripening, with marked changes in the expression of key biosynthetic genes (e.g., *GMP*, *GME* and *GGP*) across ripening stages and among cultivars [48]. For flavonoids, increases in kaempferol and apigenin aligned with previous reports of phenolic and flavonoid increases under drought [72,73]. These increases are consistent with the activation of the phenylpropanoid pathway, including the expression of genes such as *PAL* and *CHS* [74], and may also involve broader stress-responsive regulatory networks [75]. Carotenoids also showed variable responses, as described in tomato [76] and carrot [77].

When fertilizer and irrigation were simultaneously reduced, we also observed overall stability in these compounds. Thus, Zahedifar et al. [78] reported no significant differences in vitamin C across most treatments combining fertilizer and irrigation reduction in tomato. Conversely, Wang et al. [79] found higher vitamin C under the lowest input combinations. For flavonoids, kaempferol and apigenin again showed the highest number of significant differences, in line with studies showing that regulation of specific phenolics varies with the stress type and also depends on the crop and genotype [72,80]. Additionally, stable flavonoid contents under reduced irrigation and no phosphorus application have been reported in melon [81], whereas increased flavonoids under different fertilizer and irrigation inputs were observed in *Calendula officinalis* L. [82]. For carotenoids, only slight changes under combined reductions of fertilizer and irrigation were described in rocket [83], while total carotenoids decreased at the lowest doses in Swiss chard, with similar values across intermediate doses [84].

The overall stability of the evaluated compounds under low fertilizer and/or irrigation suggests that the imposed stress levels were insufficient to trigger a strong oxidative response or a generalized shift toward secondary metabolism [64,85]. This agrees with previous work that indicated that moderate deficit irrigation (around 75% of full irrigation) is a feasible water-saving strategy in pepper under Mediterranean conditions [86]. Consistently, our previous study from the same experiment showed that yield, fruit weight and fruit soluble sugars remained largely unaffected in several genotypes under low inputs [37]. This suggested that carbon supply and allocation to growth and fruit development were not substantially compromised under these moderate reductions [37,87,88]. However, limited variation in antioxidant concentrations may also reflect efficient redox homeostasis [85]. Antioxidants such as ascorbic acid, flavonoids and carotenoids participate within an integrated system in which cycling and regeneration can help maintain relatively stable basal antioxidant concentrations even when ROS signaling occurs [50]. Moreover, constitutive antioxidant capacity and acclimation ability are genotype-dependent [50,89], which may explain the stability observed in part of our collection under low-input treatments. Although plant water and nutrient status were not directly quantified, the generally stable yields and fruit weights reported in our previous work [37] suggested that a “concentration” or “dilution” effect due to changes in fruit size or biomass was unlikely to explain the largely stable antioxidant concentrations observed here [6,90]. However, changes in assimilate allocation may still contribute to variation in compound composition [90]. These results provide a useful basis for comparison with those obtained in comparable Mediterranean coastal greenhouse systems. However, extrapolating these results beyond the tested conditions should be made with caution. Further validation across seasons and locations is still needed.

PGPR inoculation has been proposed as an eco-friendly tool to enhance plant growth and fruit quality on numerous crops under stress [34,91]. However, in our study, vitamin C remained largely unchanged or even decreased in inoculated plants under reduced fertilization compared to their corresponding non-inoculated stress treatments at either ripening stage. This agreed with reports of reduced ascorbic acid under PGPR combined with stress, for example in *Brassica oleracea* var. *botrytis* under the lowest fertilization treatment [35]. Lower vitamin C may result from increased ascorbate peroxidase activity and ascorbic acid consumption during ROS detoxification [92]. Alternatively, it may derive from stress alleviation by PGPR, which could reduce ROS levels and the need for a strong antioxidant response [93]. Our findings also suggested that ripening stage modulates vitamin C responses under PGPR, supporting the work of Amor et al. [94].

Flavonoids changed only slightly under PGPR inoculation, and most genotypes maintained similar levels across stress treatments with and without inoculation. Similar flavonoid contents under fertilizer reduction and combined fertilizer stress with rhizobacteria were also described in spinach [95]. Interestingly, some genotypes in this study exhibited significant increases, highlighting Piquillo under combined fertilizer and irrigation reduction at early ripening and Isabel F1 at the fully ripe stage. Similar increases under PGPR inoculation have been reported in lettuce under reduced mineral fertilization, suggesting a possible synergistic effect on the synthesis of secondary metabolites [96]. In our work, increases in specific flavonoids may be linked to the activation of defense signaling by PGPR, including induced systemic resistance (ISR), largely mediated by jasmonic acid and other signaling components such as ethylene and salicylic acid. ISR can stimulate the phenylpropanoid pathway and the expression of genes involved in flavonoid synthesis in responsive genotypes [97]. Measuring these hormones and related molecular markers would help to confirm ISR activation.

Carotenoids showed no clear benefits from PGPR inoculation under reduced fertilization in our study. Some genotypes remained stable whereas others decreased, as also described in pepper under low fertilizer with PGPR [98]. Such reductions may reflect stress alleviation in some contexts, since bacterial priming has been associated with lower ROS signaling and a reduced induction of antioxidant defenses under stress [93]. By contrast, higher carotenoids under PGPR have been reported in lettuce under fertilizer reduction compared to non-inoculated plants under stress [34] and in pepper under lower fertilization with PGPE [98]. These contrasting results suggest that PGPR may influence carotenoid regulation by acting as biotic elicitors that modulate ROS and phytohormone pathways, including ABA signaling, which could potentially influence ripening dynamics and carotenoid biosynthesis and, therefore, final carotenoid accumulation [67,99]. Carotenoid synthesis in pepper is also sensitive to nitrogen nutrition [100]. In addition, PGPR can modify nutrient acquisition and nitrogen status [31], which may indirectly affect carotenoid accumulation through changes in plant nutritional balance and carbon allocation.

The inconsistent patterns observed between inoculated and non-inoculated plants in our study indicate genotype-dependent antioxidant variations in response to PGPR under the tested stress conditions [34,35,91,92,94]. This supported targeted application rather than a general use. PGPR performance is also influenced by plant-microbe interaction, in which root exudates seem to play a key role [101], soil properties [102], the sensitivity of bacterial strains to environmental conditions (e.g., fertilizer composition) [96], and interactions with native microbial communities [103]. Accordingly, our conclusions about PGPR effects are restricted to this single season and location, the seven pepper genotypes evaluated, the commercial PGPR consortium used and its application under reduced fertilization. The extrapolation to other seasons, environments, regimes, inoculants or pepper genotypes requires further validation.

In our trial, variability across compounds highlighted the strong influence of genotype and ripening stage on sweet pepper antioxidant profiles [6], as also reflected in the general and green-ripe ANOVAs. These results suggest that antioxidant variations to abiotic stress and PGPR under the tested conditions were complex and genotype-dependent [104,105]. At the fully ripe stage, treatment effects became more evident for vitamin C and certain flavonoids, consistent with studies that have detected a stronger environmental modulation at advanced maturity [6]. Although the magnitude of the G × T interactions varied among compounds, being less pronounced for flavonoids, the interaction patterns were consistent with the specific genotype responses observed here. Moreover, ripening involves major biochemical changes and shifts in metabolism within specific tissues in pepper that vary among genotypes [106]. These changes may partially mask or amplify treatment effects, in line with the G × R interaction detected for vitamin C and most flavonoids [6,107].

Najerano and Piquillo were included because previous work reported an interesting antioxidant profile at the fully ripe stage under organic management [6]. Piquillo has also been described as having a vigorous root system and high phosphorus uptake efficiency, with preliminary evidence of rhizosphere ammonification potential [108,109], traits that are relevant under PGPR inoculation. Thus, H1 (BGV13004-derived F1) showed the most consistent increases in vitamin C under low-input treatments at the green-ripe stage, together with BGV13004. At the fully ripe stage, Isabel F1 showed the clearest improvement in vitamin C under reduced irrigation. For flavonoids, Piquillo consistently showed high contents under reduced treatments at both ripening stages, followed by H2 (Najerano-derived F1) at the green-ripe stage. To a lesser extent, H1 increased specific flavonoids under some reduced treatments at both stages, whereas Cabañeros F1 showed higher contents of some flavonoids under combined reductions at the fully ripe stage. For carotenoids, H1 and Najerano showed the most favorable patterns under reduced fertilization and reduced irrigation, respectively. Piquillo maintained the highest overall carotenoid levels, despite specific decreases under irrigation reduction. The main increases associated with PGPR under reduced fertilization were for flavonoids in Piquillo (green-ripe) and Isabel F1 (fully ripe), compared to their corresponding non-inoculated treatments.

Overall, our main mechanistic interpretation is that the G × T and G × R interactions reflect genotype differences in constitutive antioxidant status and developmental regulation of antioxidant metabolism. These interactions also suggest differential responsiveness to moderate nutrient and water limitation [47,48,49,50,51,52,53,54,55,56,63,67,85]. Moreover, the complex and specific responses observed under PGPR inoculation suggest a redox homeostasis modulation dependent on the genotype, ripening stage and PGPR treatment, possibly involving effects on gene expression and enzyme activity related to ROS scavenging [92,93]. These responses may also depend on plant-microbe compatibility and traits associated with root characteristics, which affect nutrient uptake and hormone signaling [92,93,94,97,99,101]. Although these mechanisms were not directly assessed in the present study, our previous results from the same trial provided complementary information [37]. For instance, H1 showed antioxidant increases under stress in green fruits, although yield and fruit weight were more sensitive to reduced irrigation treatments [37]. This is compatible with antioxidant increases at early ripening in responsive genotypes [64], and it may also be related to differences in fruit size under these stress conditions [90]. By contrast, Piquillo combined elevated antioxidant contents, higher total flavonoids at the green-ripe stage under PGPR, and stable yield and fruit weight under reduced inputs [37], making it one of the most promising genotypes. Piquillo also had the smallest fruits and was the only genotype with medium mesocarp thickness (Table 7). This performance may also be related to fruit morphology, as larger and fleshy fruits have been traditionally proposed to show lower concentrations of some compounds due to a ‘dilution effect’ [6,110]. However, evidence linking fruit morphology to antioxidant concentration in pepper remains limited and inconsistent [111].

Correlation analyses provided additional insight into antioxidant coordination. At the green-ripe stage, flavonoids showed multiple positive intercorrelations [107], whereas vitamin C was largely decoupled, suggesting partially independent regulation. These associations were broadly consistent across treatment categories, indicating limited trade-offs between vitamin C and flavonoids in green fruits within our data. In fully ripe fruits, vitamin C was positively associated with carotenoids, particularly under Low-input without PGPR, but it tended to show negative associations with flavonoids, most notably with quercetin under Reduced fertilization with PGPR. These different patterns may reflect the metabolic changes related to defense signaling [47,48,50] combined with the developmental reprogramming during ripening [106]. Overall, the predominance of positive associations suggests that selecting genotypes with enhanced levels of one antioxidant class will not necessarily imply broad detrimental effects in others. However, specific antagonistic relationships may emerge under particular genotype × treatment × stage contexts.

In general terms, antioxidant responses were mainly determined by genotype and ripening stage, with more variable treatment effects. These findings support the feasibility of low-input management without major penalties in fruit nutritional quality in this collection and highlight traditional germplasm as a source of useful alleles for nutritional quality in future breeding programs [112]. Combining selected PGPR strains with well-performing genotypes could be explored as a targeted strategy, particularly for modulating flavonoids in responsive combinations under the tested conditions.

## 4. Materials and Methods

### 4.1. Plant Material

In this work, seven *Capsicum annuum* L. genotypes were evaluated, comprising three local/traditional Spanish materials, two experimental F1 hybrids developed at the COMAV Institute, and two commercial F1 hybrids (Table 7). The local and traditional genotypes were selected to represent contrasting Spanish sweet pepper morphotypes and quality attributes, including two cultivars under official EU quality schemes (PDO “Piquillo de Lodosa” and PGI “Riojano”). The experimental hybrids were new materials obtained by crossing BGV13004 or Najerano with a sweet California Wonder breeding line carrying the *L4* resistance gene. The two commercial F1 hybrids were representative of cultivars widely grown in southeastern Spain.

**Table 7:** Evaluated genotypes, descriptions, descriptive fruit traits and origin.

Accession	Description	Cultivar Type	Fruit Type	Mesocarp	Weight (g)	Origin (Provider)
BGV13004	Bell pepper	Local ecotype	Medium-elongated, triangular	Thick flesh	135 (102–167) ^2^	Spain, Basque Country
Najerano	P. G. I. Pimiento Riojano	Traditional	Medium-elongated, triangular	Thick flesh	110 (87–143)	Spain, La Rioja
Piquillo	Cons. Reg. P.D.O. Pimiento Piquillo de Lodosa	Traditional	Short conical	Medium	26 (20–31)	Spain, Navarra
H1	BGV13004 derived F1 ^1^	Experimental F1	Medium-elongated, triangular	Thick flesh	143 (107–169)	Spain, Valencia (COMAV)
H2	Najerano derived F1 ^1^	Experimental F1	Medium-elongated, triangular	Thick flesh	132 (84–178)	Spain, Valencia (COMAV)
Isabel F1	Blocky sweet pepper	Commercial F1	Blocky	Thick flesh	156 (112–203)	Spain (Ramiro Arnedo)
Cabañeros F1	Elongated sweet pepper	Commercial F1	Elongated blocky (Lamuyo)	Thick flesh	231 (219–250)	Spain (Ramiro Arnedo)

^1^ H1 and H2 were developed by crossing BGV13004 or Najerano with a red sweet California Wonder breeding line carrying the *L4* resistance gene. ^2^ Mean fruit weight and range of values. Fruit weight was previously reported in Jiménez-Pérez et al. [37].

### 4.2. Experimental Design and Pepper Cultivation Conditions

The experiment was conducted during the 2021 winter-summer season in a 400 m^2^ multi-tunnel greenhouse at the Torreblanca experimental farm (IMIDA Institute, Murcia, Spain; 37°46′34.6′′ N, 0°53′47.7′′ W). The soil at the experimental site was classified as silty loam (44.55% sand, 52.50% silt, 2.95% clay) with an alkaline pH (8.2, 1:2 soil:water at 23.9 °C). Organic matter content was 4.21%, with 2.4% organic carbon and 0.212% total nitrogen. Available macronutrients were 11.5 mg kg^−1^ phosphorus (Olsen), 317 mg kg^−1^ potassium, 23,900 mg kg^−1^ calcium, 1170 mg kg^−1^ magnesium, and 1380 mg kg^−1^ sodium. Soil parameters were obtained from an external laboratory report for site description.

Six treatments were tested, combining two fertilization levels, two irrigation levels and PGPR. The full fertilizer dose (100%) consisted of 195 kg ha^−1^ N, 162 kg ha^−1^ P_2_O_5_, 292 kg ha^−1^ K_2_O, 120 kg ha^−1^ Ca and 20 kg ha^−1^ Mg. The full irrigation regime (100%) was delivered through pressure-compensating drippers at 2.2 L h^−1^ per plant. The PGPR consisted of a commercial mix of plant growth-promoting bacteria (Bactogreen^®^, Thader Biotechnology, S.L., Murcia, Spain) containing phosphorus-solubilizing *Pseudomonas fluorescens*, potassium-solubilizing *Bacillus megaterium* and *Bacillus circulans*, and nitrogen-fixing *Azospirillum brasilense* (each ≥ 1 × 10^9^ CFU mL^−1^). PGPR was applied monthly at 3 mL plant^−1^. Due to logistical constraints and based on the composition of this PGPR mixture and the provider’s recommendations, inoculation was applied only under reduced fertilization treatments. Accordingly, PGPR responses were interpreted within the reduced fertilization context and were not extrapolated to full fertilization. Therefore, the six treatments were described in Table 8.

For the experimental design, the greenhouse was divided into two main blocks, which served as spatial replicates for possible positional heterogeneity. Each block included six cultivation rows, corresponding to the six treatments, distributed randomly. Within each row, the genotypes were also arranged randomly as subplots and included ten plants each. Thus, each genotype × treatment combination included a total of twenty plants (ten plants per block × two blocks). Plants were spaced 0.4 m within row and 1 m between rows. Cultivation followed the technical specifications for integrated pepper production established by the Order of 10 May 2012 [113].

### 4.3. Fruit Analyses

#### 4.3.1. Fruit Sampling and Preparation

Pepper fruits were collected at the two commercial ripening stages: green-ripe (final size, firm fruit, fully green) and fully ripe (firm fruit, fully red), based on the criteria used by trained technicians in commercial pepper production. For each genotype × treatment × ripening stage combination, three biological replicates were prepared. Each replicate consisted of a pooled sample of fruits harvested from four plants (two plants per block), using different plants for each replicate (i.e., sample 1: plants 2 and 3 from both blocks; sample 2: plants 5 and 6 from both blocks; sample 3: plants 8 and 9 from both blocks). A minimum of five fruits were collected per block (≥ten fruits per biological replicate). The mixture of fruits from both blocks within each replicate was done to dilute possible block/position effects. Fruits at both ripening stages could be collected from the same plants, although they were analyzed separately.

To minimize potential bias in fruit composition due to harvest timing among ripening stages and/or genotypes, fruits from all genotypes and ripening stages were sampled within the same time slot (7–11 June 2021), which coincided with the production peak of both green and fully ripe peppers. Fruits were transported to the laboratory immediately after harvest for sample preparation. Fruits were washed, the seeds were removed, and they were cut into small pieces. Each replicate was then split into two subsamples. One subsample was homogenized with a BAPI 1000 PLUS INOX domestic blender (Taurus, Oliana, Spain) to obtain a liquid extract, which was immediately aliquoted and stored at −80 °C for vitamin C analysis. The second subsample was freeze-dried in a VirTis Genesis unit (SP Scientific, Warminster, PA, USA). Samples were weighed before lyophilization to record fresh weight and again after drying to obtain dry weight. The dry material was milled with an ML130 domestic grinder (Jata, Tudela, Spain) to a fine powder and stored under dry and dark conditions until flavonoids and carotenoids analyses.

#### 4.3.2. Vitamin C

Determination of vitamin C, as the sum of ascorbic acid and dehydroascorbic acid, was based on the protocol described by Chebrolu et al. [114], with slight modifications. Vitamin C was quantified by HPLC–UV-Vis, using an Agilent 1220 Infinity LC System (Agilent Technologies, Santa Clara, CA, USA). For each sample, a liquid extract was homogenized with a vortex spin and centrifuged for 5 min at 12,000 rpm at 4 °C. The supernatant was diluted 1:4, containing 3% metaphosphoric acid (Acros Organics, Geel, Belgium), vortexed, centrifuged 5 min at 12,000 rpm and filtered with a 0.20 µm Phenex-RC 15 mm syringe filter (Phenomenex, Torrance, CA, USA). Two aliquots of the same sample were prepared, one for direct ascorbic acid quantification, and an aliquot reduced by adding an equal volume of 5 mM Tris(2-carboxyethyl)phosphine hydrochloride (Sigma-Aldrich, St. Louis, MO, USA), reducing dehydroascorbic acid to ascorbic acid to quantify total vitamin C. Chromatographic separation used a Brisa LC2 C18 column (3 µm; 150 × 4.6 mm; Teknokroma, Barcelona, Spain) at 254 nm. The analysis was performed under isocratic conditions for 15 min using a mobile phase of 5% methanol and 95% HPLC-grade water at 1% acetic acid. The injection volume was 5 µL, and the flow rate was 1.0 mL min^−1^. Quantification was based on a calibration curve for the external standard of ascorbic acid (99%) (Sigma-Aldrich). Ascorbic acid and vitamin C (ascorbic acid + dehydroascorbic acid) were expressed as mg kg^−1^ fresh weight (fw). Because ascorbic acid showed similar magnitudes and trends to vitamin C, only vitamin C is presented in the main text; ascorbic acid values are available in Tables S1–S4.

#### 4.3.3. Flavonoids

The main flavonoids in pepper were analyzed using a method based on Bae et al. [115]. Determination of quercetin, luteolin, kaempferol and apigenin was performed by HPLC–UV-Vis, using a 1220 Infinity LC System (Agilent Technologies). For flavonoids extraction, one sample of 0.1 g per lyophilized replicate was mixed with 1.5 mL of extraction solution of methanol: Milli-Q^®^ water (80:20, *v*/*v*) with 0.1% (*w*/*v*) of 2,3-tert-butyl-4-hydroxyanisole (BHT). Each sample was homogenized, incubated in an Elmasonic Select ultrasonic bath (Elma, Hohentwiel, Germany) 1h at 40˚C and centrifuged at 10,000 rpm for 5 min. Then, the acid hydrolysis of flavonoid glucosides to the aglycone form was performed treating the supernatant with 3 M HCl (2:1) in a multiplace dry block heater (J.P. SELECTA^®^, Barcelona, Spain) at 95˚C for 1h. The hydrolyzed replicate was centrifuged at 7000 rpm for 5 min and filtered with a 0.20 µm Econofltr PTFE 13 mm syringe filter (Agilent Technologies). The main flavonoids were separated using a Brisa LC2 C18 column (3 µm; 150 × 4.6 mm; Teknokroma) at 360 nm. Column temperature was set to 30 °C and injection volume was 10 µL. The mobile phase consisted of HPLC-grade water at 0.1% formic acid (A) and HPLC-grade methanol at 0.1% formic acid (B). A gradient elution for 20 min, with a flow rate of 0.8 mL min^−1^, was used as follows: a linear gradient of 40–100% B (0–10 min), 100% B (10–15 min), and a linear gradient of 40–100% B (15–20 min). Flavonoid quantification consisted of performing calibration curves for external standards of quercetin (≥95%), luteolin (98%), kaempferol (97%) and apigenin (95%) (Sigma-Aldrich). Total flavonoids were calculated as the sum of the four individual flavonoids and were expressed as mg kg^−1^ fw.

#### 4.3.4. Carotenoids

For carotenoids analysis, a spectrophotometric method described by Hornero-Méndez and Mínguez-Mosquera, with some modifications [116], was used. Red carotenoids, yellow–orange carotenoids and total carotenoids were analyzed by spectrophotometry, using a UviLine 9400 UV–vis spectrophotometer (SCHOTT Instruments, Mainz, Germany). Carotenoid extraction was performed using 0.1 g of each lyophilized replicate in 20 mL of acetone (ITW Reagents, Monza, Italy), by orbital shaking using a Vibrax shaker (OVAN, Barcelona, Spain) for 1h at 200 rpm, maintaining the samples in the dark. Each sample was filtered with a 0.11 µm FILTER-LAB^®^ filter paper (Filtros Anoia, Barcelona, Spain), and acetone was added to 25 mL. Carotenoids were quantified by measuring the absorbance values at 472 nm (Δ472) and 508 nm (Δ508) and applying the following formulas:

$$Red\;carotenoids\;\left(µg\;{mL}^{-1}\right)=\;\frac{∆508\cdot 2144-∆472\cdot 403.3}{270.9}$$

$$Yellow-orange\;carotenoids\;\left(µg\;{mL}^{-1}\right)=\frac{∆472\cdot 1724.3-∆508\cdot 2450.1}{270.9}$$

Total carotenoids were calculated as the sum of red carotenoids and yellow-orange carotenoids. Concentrations were expressed as mg kg^−1^ fw.

#### 4.3.5. Statistical Analysis

For each genotype × treatment combination, values were averaged at both ripening stages. Normality was evaluated using the Kolmogorov–Smirnov test for global comparisons (*n* > 50) and the Shapiro–Wilk test for comparisons within each genotype × treatment and treatment × genotype subset at each ripening stage (*n* < 50) [117], using Statgraphics Centurion 18 (StatPoint Technologies, Warrenton, VA, USA). When necessary, datasets were transformed to better meet ANOVA assumptions.

A general three-way ANOVA was fitted with genotype (G; the seven studied genotypes), treatment (T; the six combinations of fertilizer dose, irrigation dose and PGPR inoculation) and ripening stage (R; green-ripe and fully ripe) as main factors, including all two-way interactions. In addition, separate two-way ANOVAs (factors G, T, G × T) were performed at each ripening stage. The relative contributions of the factors were interpreted from the ANOVA mean squares. Overall means for genotype and treatment were reported as least-squares means (LS means) with their LS standard errors (LS SE). Mean separation was performed using Duncan’s post hoc test at *p* < 0.05, based on the ANOVA residual mean square. Subsequently, simple effect comparisons were also performed among genotypes within each treatment and among treatments within each genotype. Given the large number of comparisons in this multi-factor experiment, Duncan’s test was used as an exploratory mean separation procedure to identify genotype × treatment patterns and was interpreted in conjunction with the ANOVA. Because PGPR was applied only under reduced fertilization, PGPR effects were evaluated by comparison with the corresponding non-inoculated stress treatments. Comparisons with the control were also reported as a descriptive reference baseline to provide a common reference across treatments.

Correlations between antioxidant traits were evaluated using Spearman’s rank correlation coefficient (*ρ*) in R (version 4.5.2; RStudio, Posit Software, Boston, MA, USA). Analyses were performed separately for each ripening stage: vitamin C and flavonoids at the green-ripe stage, and vitamin C, flavonoids and carotenoids at the fully ripe stage (*n* = 126 per stage). Additionally, analyses were repeated within three treatment categories: control (100F + 100I), low-input treatments without PGPR (50F + 100I, 100F + 75I, 50F + 75I) and reduced fertilization with PGPR (50F + 100I + PGPR, 50F + 75I + PGPR) (Figure S1). The *p*-values were adjusted for multiple testing using the Benjamini–Hochberg false discovery rate (FDR) procedure.

## 5. Conclusions

Genotype and ripening stage were the main determinants of the antioxidant profile, whereas treatment effects depended more on the evaluated compound, genotype and ripening stage. Under the tested stress conditions, several genotypes maintained largely stable vitamin C, flavonoids and carotenoids, consistent with a limited influence of these input reductions. Some traditional genotypes and related experimental hybrids (e.g., Piquillo and H1) performed similarly or, for specific compounds and stages, better under stress than in control conditions.

PGPR inoculation did not provide consistent benefits for vitamin C or carotenoids under reduced fertilization, and in some genotype × treatment combinations these traits decreased. By contrast, PGPR effects were mainly observed for flavonoids, with increases in total and/or specific flavonoids in selected genotypes and ripening stages. Therefore, under this specific season, location, the genotype collection and inoculant tested, this PGPR consortium does not appear suitable as a general strategy to improve the antioxidant profile under reduced fertilization. Its potential seems to lie in targeted genotype-inoculant combinations for modulating flavonoids under the evaluated conditions.

Correlation analyses suggested limited overall trade-offs among antioxidant compounds. However, vitamin C was the main exception, showing negative associations with flavonoids at the fully ripe stage, most notably with quercetin.

From a breeding perspective, these findings highlight the value of screening and selecting materials from local and traditional Spanish germplasm as valuable sources of fruit nutritional quality under more sustainable pepper production systems in southeastern Spain. Future research should include multi-season and multi-location validation, testing PGPR inoculation at both fertilization levels and evaluating additional PGPR × genotype combinations under other suboptimal conditions. In addition, it would be valuable to include the assessment of postharvest traits (e.g., physiological disorders, shelf-life performance), as well as direct measurements of plant water and nutrient status.

## Figures and Tables

**Table 1:** General three-way ANOVA and individual two-way ANOVAs at green-ripe and fully ripe stages for vitamin C (VC), quercetin (QUE), luteolin (LUT), kaempferol (KAE), apigenin (API) and total flavonoids (TF). Individual ANOVA for fully ripe stage for red carotenoids (RC), yellow-orange carotenoids (Y-OC) and total carotenoids (TC).

General ANOVA		VC			QUE	LUT	KAE	API	TF			RC	Y-OC	TC
Effect	df ^1^	MS ^2^		df	MS	MS	MS	MS	MS		df	MS	MS	MS
Main effect														
Genotype (G)	6	695709 ***		6	0.08 ***	0.70 ***	0.04 *	0.41 ***	2.15 ^3^ ***					
Treatment (T)	5	541725 ***		5	0.08 ***	0.03 **	0.13 ***	0.12 ***	0.31 **					
Ripening stage (R)	1	1.7E6 ***		1	0.26 ***	0.11 ***	17.38 ***	5.91 ***	1.11 ***					
Interactions														
G × T	30	67829 **		30	0.03 *	0.01 ^NS^	0.03 **	0.01 **	0.13 ^NS^					
G × R	6	181431 ***		6	0.02 ^NS^	0.03 **	0.06 **	0.03 ***	0.25 *					
T × R	5	595963 ***		5	0.10 ***	0.02 *	0.09 ***	0.03 ***	0.35 **					
Error	198	34889		198	0.02	0.01	0.02	0.00	0.09					
**Green-ripe stage ANOVA**														
Main effect														
Genotype (G)	6	683125 ***		6	0.06 **	0.47 ***	0.01 *	0.31 ***	0.18 ***					
Treatment (T)	5	385786 ***		5	0.06 *	0.05 **	0.02 ***	0.12 ***	0.03 **					
Interactions														
G × T	30	74184 **		30	0.03 *	0.02 ^NS^	0.00 ^NS^	0.01 **	0.02 *					
Error	84	34811		84	0.02	0.01	0.00	0.01	0.01					
**Fully ripe stage ANOVA**														
Main effect														
Genotype (G)	6	194016 ***		6	18.81 *	0.26 ***	0.30 **	0.13 ***	0.09 ***		6	17859 ***	5553 ***	41741 ***
Treatment (T)	5	751902 ***		5	52.51 ***	0.01 ^NS^	0.85 ***	0.03 ***	0.02 **		5	3065 ***	142 ^NS^	3483 ***
Interactions														
G × T	30	81913 ***		30	10.00 ^NS^	0.01 ^NS^	0.21 ***	0.00 *	0.01 ^NS^		30	1668 ***	305 ***	3267 **
Error	84	15904		84	7.10	0.01	0.08	0.00	0.01		84	622	117	1206

^1^ df: degrees of freedom. ^2^ MS: mean square (values are shown rounded for readability). ^NS^, *, ** and *** indicate non-significant (*p* ≥ 0.05) and significant at *p* < 0.05, *p* < 0.01 and *p* < 0.001, respectively, based on ANOVA F-tests. ^3^ For TF, MS values in the general ANOVA are shown as MS × 10^3^ for presentation.

**Table 2:** Mean content of total vitamin C in green-ripe pepper fruits across the evaluated genotypes and treatments.

Vitamin C (mg kg^−1^)																		
Genotype	100F + 100I			50F + 100I			50F + 100I + PGPR			100F + 75I			50F + 75I			50F + 75I + PGPR		
BGV13004	714	ab ^1^	AB ^2^	1250	e	CD	572	abc	A	1442	d	D	984	b	BC	929	b	ABC
Najerano	507	a	A	461	ab	A	514	ab	A	907	ab	B	638	a	AB	572	a	A
Piquillo	1010	ab	A	925	cd	A	714	bc	A	980	b	A	1022	b	A	781	ab	A
H1	747	ab	A	1103	de	BC	801	bc	AB	1309	cd	C	1296	c	C	1215	c	C
H2	968	ab	A	733	bc	A	878	c	A	1044	bc	A	915	ab	A	701	ab	A
Isabel F1	1168	b	A	852	cd	A	895	c	A	1150	bcd	A	993	b	A	860	ab	A
Cabañeros F1	621	a	AB	435	a	AB	317	a	A	644	a	AB	787	ab	B	666	ab	AB

Data are the mean of three replicates. ^1^ Different lowercase letters indicate significant differences among genotypes within a treatment; ^2^ different uppercase letters indicate significant differences among treatments within a genotype (Duncan’s test, *p* < 0.05).

**Table 3:** Mean content of total vitamin C in fully ripe pepper fruits across the evaluated genotypes and treatments.

Vitamin C (mg kg^−1^)																		
Genotype	100F + 100I			50F + 100I			50F + 100I + PGPR			100F + 75I			50F + 75I			50F + 75I + PGPR		
BGV13004	1602	d ^1^	C ^2^	1203	bc	B	1317	c	B	949	ab	A	921	b	A	800	c	A
Najerano	1021	a	AB	1003	b	AB	1039	ab	AB	1144	b	B	1008	bc	AB	871	c	A
Piquillo	1243	c	B	1261	c	B	1115	abc	AB	862	a	A	1077	bc	AB	893	c	A
H1	1177	abc	BC	1325	c	C	1111	abc	BC	1068	ab	B	990	bc	B	635	abc	A
H2	1065	ab	B	1173	bc	B	972	ab	B	1073	ab	B	650	a	A	678	bc	A
Isabel F1	1181	bc	B	1188	bc	B	1197	bc	B	1468	c	C	1143	c	B	443	ab	A
Cabañeros F1	1083	abc	D	766	a	B	941	a	C	897	a	BC	978	bc	CD	349	a	A

Data are the mean of three replicates. ^1^ Different lowercase letters indicate significant differences among genotypes within a treatment; ^2^ different uppercase letters indicate significant differences among treatments within a genotype (Duncan’s test, *p* < 0.05).

**Table 4:** Mean content of quercetin, luteolin, kaempferol, apigenin and total flavonoids in green-ripe pepper fruits across the evaluated genotypes and treatments.

Quercetin (mg kg^−1^)																		
Genotype	100F + 100I			50F + 100I			50F + 100I + PGPR			100F + 75I			50F + 75I			50F + 75I + PGPR		
BGV13004	12.69	a ^1^	A ^2^	10.39	ab	A	9.85	ab	A	6.11	a	A	12.34	ab	A	5.60	ab	A
Najerano	9.45	a	AB	9.79	ab	AB	9.99	ab	AB	11.54	b	AB	14.53	b	B	8.67	bcd	A
Piquillo	11.52	a	ABC	13.32	b	BC	12.04	ab	ABC	8.43	ab	A	10.35	ab	AB	15.83	d	C
H1	9.63	a	A	13.66	b	A	12.64	ab	A	9.00	ab	A	8.39	ab	A	9.57	cd	A
H2	8.85	a	B	16.06	b	C	14.33	b	C	8.29	ab	B	11.53	ab	BC	5.30	a	A
Isabel F1	7.64	a	A	6.90	a	A	7.93	a	A	8.27	ab	A	7.69	a	A	8.55	abc	A
Cabañeros F1	11.60	a	A	9.83	ab	A	9.85	ab	A	8.18	ab	A	15.46	b	A	12.29	cd	A
**Luteolin (mg kg** **^−1^** **)**																		
BGV13004	12.69	bc	A	10.79	b	A	11.54	b	A	7.76	ab	A	12.74	ab	A	7.98	ab	A
Najerano	5.82	a	A	7.71	a	AB	7.71	a	AB	6.75	a	AB	8.18	a	AB	9.65	bc	B
Piquillo	20.46	c	A	21.09	c	A	21.23	c	A	16.60	d	A	19.71	c	A	38.59	d	B
H1	8.76	ab	A	9.08	ab	A	11.55	ab	A	9.82	bc	A	11.81	ab	A	10.64	bc	A
H2	7.41	ab	AB	11.12	b	BC	9.91	ab	ABC	6.92	ab	A	12.42	ab	C	6.76	a	A
Isabel F1	8.36	ab	A	7.39	a	A	8.92	ab	A	5.98	a	A	8.30	a	A	8.94	ab	A
Cabañeros F1	12.22	abc	A	11.47	b	A	12.48	b	A	13.49	cd	A	15.50	c	A	16.68	cd	A
**Kaempferol (mg kg** **^−1^** **)**																		
BGV13004	0.60	a	AB	0.78	a	AB	0.74	ab	AB	0.72	bc	AB	0.96	a	B	0.53	a	A
Najerano	0.57	a	A	0.55	a	A	0.55	a	A	0.72	bc	A	0.76	a	A	0.68	b	A
Piquillo	0.65	a	B	0.58	a	AB	0.62	ab	AB	0.49	a	A	0.73	a	BC	1.06	cd	C
H1	0.55	a	A	0.66	a	AB	0.71	ab	AB	0.62	abc	AB	0.98	a	B	0.75	bc	AB
H2	0.50	a	A	0.75	a	BC	0.91	b	C	0.82	c	BC	0.93	a	C	0.60	ab	AB
Isabel F1	0.55	a	AB	0.40	a	A	0.57	a	AB	0.51	ab	A	0.76	a	BC	1.00	cd	C
Cabañeros F1	1.07	a	A	0.65	a	A	0.72	ab	A	0.70	abc	A	1.12	a	A	1.34	d	A
**Apigenin (mg kg** **^−1^** **)**																		
BGV13004	1.11	b	A	1.17	a	AB	1.25	c	AB	1.27	b	AB	1.30	a	B	1.19	a	AB
Najerano	0.99	ab	A	1.05	a	AB	1.08	ab	BC	1.25	b	D	1.19	a	CD	1.17	a	CD
Piquillo	1.71	c	A	1.89	b	A	1.75	d	A	1.71	c	A	2.12	b	B	2.62	b	C
H1	0.93	a	A	1.16	a	B	1.18	bc	B	1.28	b	BC	1.48	a	C	1.39	a	C
H2	1.02	ab	A	1.07	a	A	1.16	bc	AB	1.13	a	AB	1.65	a	B	1.21	a	AB
Isabel F1	1.13	b	ABC	1.08	a	AB	1.04	a	A	1.04	a	A	1.19	a	BC	1.26	a	C
Cabañeros F1	1.06	ab	A	1.14	a	AB	1.04	a	A	1.36	b	B	1.26	a	AB	1.43	a	B
**Total flavonoids (mg kg** **^−1^** **)**																		
BGV13004	27.08	ab	A	23.13	bc	A	23.38	abc	A	15.86	a	A	27.35	ab	A	15.30	ab	A
Najerano	16.83	a	A	19.09	ab	A	19.33	ab	A	20.26	abc	A	24.65	ab	A	20.17	bc	A
Piquillo	34.34	b	A	36.88	c	A	35.64	d	A	27.23	c	A	32.91	b	A	58.10	c	B
H1	19.87	ab	A	24.55	bc	A	26.07	bcd	A	20.72	abc	A	22.66	ab	A	22.34	c	A
H2	17.78	ab	B	29.00	c	C	26.31	cd	C	17.14	ab	B	26.54	ab	C	13.86	a	A
Isabel F1	17.68	ab	A	15.77	a	A	18.46	a	A	15.80	a	A	17.93	a	A	19.75	bc	A
Cabañeros F1	25.95	ab	A	23.09	bc	A	24.08	abcd	A	23.72	bc	A	33.30	b	A	31.74	cd	A

Data are the mean of three replicates. ^1^ Different lowercase letters indicate significant differences among genotypes within a treatment; ^2^ different uppercase letters indicate significant differences among treatments within a genotype (Duncan’s test, *p* < 0.05).

**Table 5:** Mean content of quercetin, luteolin, kaempferol, apigenin and total flavonoids in fully ripe pepper fruits across the evaluated genotypes and treatments.

Quercetin (mg kg^−1^)																		
Genotype	100F + 100I			50F + 100I			50F + 100I + PGPR			100F + 75I			50F + 75I			50F + 75I + PGPR		
BGV13004	6.80	a ^1^	A ^2^	9.09	a	A	5.88	a	A	7.46	ab	A	10.19	ab	A	10.01	a	A
Najerano	5.85	a	A	10.95	a	AB	8.79	a	AB	10.93	b	AB	7.47	ab	AB	13.34	a	B
Piquillo	11.45	b	B	11.05	a	B	6.05	a	A	6.56	a	A	12.24	ab	B	13.01	a	B
H1	7.75	ab	A	8.59	a	A	6.58	a	A	8.43	ab	A	8.62	ab	A	9.95	a	A
H2	7.31	ab	A	12.98	a	B	7.35	a	A	8.06	ab	AB	13.25	b	AB	11.29	a	AB
Isabel F1	7.80	ab	A	7.29	a	A	6.47	a	A	6.38	a	A	5.85	a	A	13.36	a	B
Cabañeros F1	6.22	a	A	8.23	a	A	9.11	a	A	6.62	a	A	8.25	ab	A	7.96	a	A
**Luteolin (mg kg** **^−1^** **)**																		
BGV13004	9.53	ab	A	11.90	a	A	10.56	abc	A	12.12	b	A	12.44	bc	A	13.25	a	A
Najerano	9.08	a	A	9.63	a	A	9.58	ab	A	7.83	a	A	6.96	a	A	8.98	a	A
Piquillo	22.10	c	AB	22.05	b	AB	14.91	d	A	21.12	c	AB	21.69	d	A	24.03	b	B
H1	11.72	b	A	12.40	a	A	11.94	bcd	A	12.81	b	A	11.48	bc	A	13.25	a	A
H2	10.31	ab	A	10.00	a	A	8.84	a	A	10.25	ab	A	9.83	b	A	10.50	a	A
Isabel F1	10.18	ab	A	10.52	a	A	10.36	abc	A	9.96	ab	A	9.73	bc	A	11.12	a	A
Cabañeros F1	9.60	ab	A	12.45	a	AB	13.66	cd	AB	11.87	b	AB	14.33	cd	B	10.22	a	AB
**Kaempferol (mg kg** **^−1^** **)**																		
BGV13004	1.81	bc	AB	1.52	ab	A	2.10	ab	ABC	2.46	b	C	2.19	a	BC	1.82	a	AB
Najerano	1.63	ab	A	1.55	ab	A	2.43	b	B	2.27	b	B	1.69	a	A	2.39	b	B
Piquillo	2.08	c	A	2.00	b	A	1.79	a	A	1.61	a	A	1.70	a	A	2.11	ab	A
H1	2.13	c	AB	1.79	ab	A	2.12	ab	AB	2.35	b	B	1.68	a	A	1.90	a	AB
H2	2.05	bc	A	1.67	ab	A	2.04	ab	A	2.04	ab	A	1.54	a	A	2.00	a	A
Isabel F1	1.20	a	A	1.33	a	A	1.94	ab	C	2.01	ab	C	1.58	a	B	1.95	a	C
Cabañeros F1	1.33	a	A	1.32	a	A	2.30	ab	B	1.70	a	AB	2.04	a	B	1.73	a	AB
**Apigenin (mg kg** **^−1^** **)**																		
BGV13004	1.95	b	A	1.76	a	A	1.78	a	A	2.27	c	B	2.04	ab	AB	1.97	a	A
Najerano	1.93	b	C	1.71	a	AB	1.65	a	A	1.94	b	C	1.84	a	ABC	1.89	a	BC
Piquillo	3.22	c	ABC	2.97	b	AB	2.60	b	A	3.76	d	C	3.24	c	BC	3.34	b	BC
H1	1.75	ab	A	1.96	a	ABC	1.80	a	AB	2.16	c	C	2.20	b	C	2.01	a	BC
H2	1.68	ab	A	1.66	a	A	1.71	a	A	2.19	c	B	1.88	a	AB	1.93	a	AB
Isabel F1	2.05	b	B	1.74	a	AB	1.65	a	A	1.98	b	AB	2.10	ab	B	2.04	a	B
Cabañeros F1	1.60	a	A	1.72	a	AB	1.76	a	AB	1.68	a	A	2.05	ab	C	1.95	a	BC
**Total flavonoids (mg kg** **^−1^** **)**																		
BGV13004	20.10	a	A	24.27	a	A	20.32	a	A	24.30	ab	A	26.86	abc	A	27.04	ab	A
Najerano	18.48	a	A	23.85	a	A	22.45	a	A	22.97	a	A	17.96	a	A	26.60	a	A
Piquillo	38.86	b	B	38.07	b	B	25.34	a	A	33.06	b	AB	38.88	c	B	42.49	b	B
H1	23.35	a	A	24.74	a	A	22.44	a	A	25.75	ab	A	23.97	abc	A	27.11	ab	A
H2	21.36	a	A	26.31	ab	A	19.94	a	A	22.54	ab	A	26.50	abc	A	25.72	a	A
Isabel F1	21.22	a	A	20.88	a	A	20.42	a	A	20.33	a	A	19.26	ab	A	28.48	ab	B
Cabañeros F1	18.75	a	A	23.73	a	A	26.83	a	A	21.87	a	A	26.67	bc	A	21.86	a	A

Data are the mean of three replicates. ^1^ Different lowercase letters indicate significant differences among genotypes within a treatment; ^2^ different uppercase letters indicate significant differences among treatments within a genotype (Duncan’s test, *p* < 0.05).

**Table 6:** Mean content of red, yellow-orange and total carotenoids in fully ripe pepper fruits across the evaluated genotypes and treatments.

Red Carotenoids (mg kg^−1^)																		
Genotype	100F + 100I			50F + 100I			50F + 100I + PGPR			100F + 75I			50F + 75I			50F + 75I + PGPR		
BGV13004	219	c ^1^	C ^2^	178	cd	BC	165	c	AB	126	ab	A	155	bcd	AB	152	bc	AB
Najerano	147	b	AB	122	ab	A	150	bc	AB	166	ab	B	130	bc	AB	155	bc	AB
Piquillo	222	c	C	221	de	C	153	bc	A	167	b	AB	195	d	BC	191	c	B
H1	164	b	A	234	e	B	173	c	A	151	ab	A	168	cd	A	132	ab	A
H2	170	b	A	174	cd	A	131	ab	A	135	ab	A	115	ab	A	147	b	A
Isabel F1	106	a	AB	141	bc	B	126	ab	AB	118	ab	AB	122	abc	AB	92	a	A
Cabañeros F1	107	a	A	79	a	A	120	a	A	98	a	A	77	a	A	119	ab	A
**Yellow–Orange carotenoids (mg kg** **^−1^** **)**																		
BGV13004	65	d	B	48	abc	AB	47	ab	AB	33	a	A	54	bc	AB	42	bc	AB
Najerano	43	bc	A	39	ab	A	44	ab	A	72	b	B	49	abc	A	53	c	A
Piquillo	87	e	AB	97	d	B	67	d	A	70	b	A	96	d	B	82	d	AB
H1	52	cd	AB	74	cd	B	61	cd	AB	57	ab	AB	64	c	AB	42	bc	A
H2	56	cd	A	53	bc	A	50	bc	A	57	ab	A	46	abc	A	52	c	A
Isabel F1	29	ab	AB	41	ab	B	34	a	AB	40	a	B	37	ab	AB	23	a	A
Cabañeros F1	23	a	A	22	a	A	36	a	A	35	a	A	28	a	A	35	b	A
**Total carotenoids (mg kg** **^−1^** **)**																		
BGV13004	284	c	B	225	b	AB	212	bc	A	159	ab	A	209	bc	A	194	b	A
Najerano	190	b	AB	161	ab	A	193	ab	AB	238	b	B	179	bc	A	207	b	AB
Piquillo	309	c	C	318	c	C	220	bc	A	237	b	AB	291	d	C	274	c	BC
H1	216	b	A	308	c	B	234	c	A	208	ab	A	232	cd	A	174	b	A
H2	226	b	A	227	b	A	182	ab	A	192	ab	A	161	ab	A	199	b	A
Isabel F1	134	a	AB	182	b	B	160	a	AB	157	ab	AB	160	ab	AB	116	a	A
Cabañeros F1	130	a	A	100	a	A	156	a	A	133	a	A	105	a	A	155	ab	A

Data are the mean of three replicates. ^1^ Different lowercase letters indicate significant differences among genotypes within a treatment; ^2^ different uppercase letters indicate significant differences among treatments within a genotype (Duncan’s test, *p* < 0.05).

**Table 8:** Treatment codes and descriptions.

Treatment Code	Fertilizer Dose (%)	Irrigation Dose (%)	PGPR Inoculation
100F + 100I (Control)	100	100	No
50F + 100I	50	100	No
50F + 100I + PGPR	50	100	Yes
100F + 75I	100	75	No
50F + 75I	50	75	No
50F + 75I + PGPR	50	75	Yes

## Data Availability

The raw data supporting the conclusions of this article will be made available by the authors on request.

## Supplementary Materials

The following supporting information can be downloaded from: https://www.mdpi.com/article/10.3390/plants15081278/s1; Table S1. Mean content of ascorbic acid (AA), vitamin C (VC), quercetin (QUE), luteolin (LUT), kaempferol (KAE), apigenin (API), total flavonoids (TF), red carotenoids (RC), yellow-orange carotenoids (Y-OC) and total carotenoids (TC) in green-ripe and fully ripe pepper fruits across the evaluated genotypes within all treatments; Table S2. Mean content of ascorbic acid (AA), vitamin C (VC), quercetin (QUE), luteolin (LUT), kaempferol (KAE), apigenin (API), total flavonoids (TF), red carotenoids (RC), yellow-orange carotenoids (Y-OC) and total carotenoids (TC) in green-ripe and fully ripe pepper fruits across the evaluated treatments within all genotypes; Table S3. Mean content of ascorbic acid and total vitamin C in green-ripe pepper fruits across the evaluated genotypes and treatments; Table S4. Mean content of ascorbic acid and total vitamin C in fully ripe pepper fruits across the evaluated genotypes and treatments; Table S5. Mean content of quercetin, luteolin, kaempferol, apigenin and total flavonoids in green-ripe pepper fruits across the evaluated genotypes and treatments; Table S6. Mean content of quercetin, luteolin, kaempferol, apigenin and total flavonoids in fully ripe pepper fruits across the evaluated genotypes and treatments; Table S7. Mean content of red, yellow-orange and total carotenoids in fully ripe pepper fruits across the evaluated genotypes and treatments. Figure S1. Spearman’s rank correlation (*ρ*) heatmap for green-ripe stage: (a) control, (b) low-input without PGPR and (c) reduced fertilization with PGPR categories; and for the fully ripe stage: (d) control, (e) low-input without PGPR and (f) reduced fertilization with PGPR categories. *, ** and *** indicate *p* < 0.05, 0.01 and 0.001, respectively.
