# Supplementary material for: Effect of Fertilization, Irrigation and Microbial Biostimulant on the Antioxidant Profile of Some Sweet Pepper Genotypes
Source: Plants (Basel). 2026 Apr 21;15(8):1278. doi: 10.3390/plants15081278 (PMC13120125; doi:10.3390/plants15081278)
Supplement: Supplementary file 1 [file plants-15-01278-s001.zip › plants-4220104-supplementary-revised.pdf]

Table S1. Mean content of ascorbic acid (AA), vitamin C (VC), quercetin (QUE), luteolin (LUT), kaempferol (KAE), apigenin (API), total flavonoids (TF), red carotenoids (RC), yellow-orange carotenoids (Y-OC) and total carotenoids (TC) in green-ripe and fully ripe pepper fruits of the evaluated genotypes within all treatments.

Genotype	AA (mg kg^{-1})		VC (mg kg^{-1})		QUE (mg kg^{-1})		LUT (mg kg^{-1})		KAE (mg kg^{-1})		API (mg kg^{-1})		TF (mg kg^{-1})		RC (mg kg^{-1})		Y-OC (mg kg^{-1})		TC (mg kg^{-1})	
**Green-ripe stage**																				
BGV13004	855 ± 49	BC	982 ± 44	BC	9.50 ± 0.87	AB	10.58 ± 0.74	B	0.72 ± 0.06	A	1.21 ± 0.03	B	22.02 ± 1.47	B						
Najerano	456 ± 49	A	600 ± 44	A	10.66 ± 0.87	BC	7.64 ± 0.74	A	0.64 ± 0.06	A	1.12 ± 0.03	A	20.06 ± 1.47	AB						
Piquillo	598 ± 49	A	905 ± 44	B	11.92 ± 0.87	C	22.95 ± 0.74	D	0.69 ± 0.06	A	1.97 ± 0.03	C	37.52 ± 1.47	D						
H1	966 ± 49	C	1078 ± 44	C	10.48 ± 0.87	BC	10.28 ± 0.74	B	0.71 ± 0.06	A	1.24 ± 0.03	B	22.70 ± 1.47	B						
H2	804 ± 49	B	873 ± 44	B	10.73 ± 0.87	BC	9.09 ± 0.74	AB	0.75 ± 0.06	AB	1.21 ± 0.03	AB	21.77 ± 1.47	B						
Isabel F1	972 ± 49	C	986 ± 44	BC	7.83 ± 0.87	A	7.98 ± 0.74	A	0.63 ± 0.06	A	1.12 ± 0.03	A	17.57 ± 1.47	A						
Cabañeros F1	504 ± 49	A	578 ± 44	A	11.20 ± 0.87	BC	13.63 ± 0.74	C	0.93 ± 0.06	B	1.22 ± 0.03	AB	26.98 ± 1.47	C						
**Fully ripe stage**																				
BGV13004	1112 ± 30	D	1132 ± 30	D	8.24 ± 0.63	AB	11.63 ± 0.59	CD	1.98 ± 0.07	C	1.96 ± 0.04	C	23.81 ± 1.18	A	166 ± 6	D	48 ± 3	B	214 ± 8	BC
Najerano	970 ± 30	BC	1014 ± 30	BC	9.55 ± 0.63	AB	8.68 ± 0.59	A	1.99 ± 0.07	C	1.83 ± 0.04	AB	22.05 ± 1.18	A	145 ± 6	C	50 ± 3	B	195 ± 8	B
Piquillo	1038 ± 30	CD	1075 ± 30	CD	10.06 ± 0.63	B	20.98 ± 0.59	E	1.88 ± 0.07	BC	3.19 ± 0.04	D	36.12 ± 1.18	B	191 ± 6	E	83 ± 3	D	275 ± 8	D
H1	1041 ± 30	CD	1051 ± 30	CD	8.32 ± 0.63	AB	12.27 ± 0.59	D	2.00 ± 0.07	C	1.98 ± 0.04	C	24.56 ± 1.18	A	170 ± 6	D	58 ± 3	C	229 ± 8	C
H2	932 ± 30	B	935 ± 30	B	10.04 ± 0.63	B	9.96 ± 0.59	B	1.89 ± 0.07	BC	1.84 ± 0.04	AB	23.73 ± 1.18	A	145 ± 6	C	52 ± 3	BC	198 ± 8	B
Isabel F1	1103 ± 30	D	1103 ± 30	CD	7.86 ± 0.63	A	10.31 ± 0.59	BC	1.67 ± 0.07	A	1.93 ± 0.04	BC	21.76 ± 1.18	A	118 ± 6	B	34 ± 3	A	151 ± 8	A
Cabañeros F1	816 ± 30	A	836 ± 30	A	7.73 ± 0.63	A	12.02 ± 0.59	D	1.74 ± 0.07	AB	1.79 ± 0.04	A	23.28 ± 1.18	A	100 ± 6	A	30 ± 3	A	130 ± 8	A

Data are the mean of eighteen replicates (LS Mean ± LS SE). Different uppercase letters indicate significant differences among genotypes according to the Duncan test ($p < 0.05$).

Table S2. Mean content of ascorbic acid (AA), vitamin C (VC), quercetin (QUE), luteolin (LUT), kaempferol (KAE), apigenin (API), total flavonoids (TF), red carotenoids (RC), yellow-orange carotenoids (Y-OC) and total carotenoids (TC) in green-ripe and fully ripe pepper fruits of the evaluated treatments within all genotypes.

Genotype	AA (mg kg^{-1})		VC (mg kg^{-1})		QUE (mg kg^{-1})		LUT (mg kg^{-1})		KAE (mg kg^{-1})		API (mg kg^{-1})		TF (mg kg^{-1})		RC (mg kg^{-1})		Y-OC (mg kg^{-1})		TC (mg kg^{-1})	
**Green-ripe stage**																				
100F + 100I	723 ± 46	BC	819 ± 41	B	10.20 ± 0.80	AB	10.82 ± 0.68	AB	0.64 ± 0.05	A	1.13 ± 0.03	A	22.79 ± 1.37	AB						
50F + 100I	724 ± 46	BC	823 ± 41	B	11.42 ± 0.80	B	11.23 ± 0.68	BC	0.62 ± 0.05	A	1.22 ± 0.03	B	24.50 ± 1.37	BC						
50F + 100I + PGPR	517 ± 46	A	670 ± 41	A	10.95 ± 0.80	B	11.90 ± 0.68	BC	0.69 ± 0.05	A	1.21 ± 0.03	B	24.75 ± 1.37	BC						
100F + 75I	979 ± 46	D	1068 ± 41	D	8.55 ± 0.80	A	9.62 ± 0.68	A	0.65 ± 0.05	A	1.29 ± 0.03	C	20.11 ± 1.37	A						
50F + 75I	825 ± 46	C	948 ± 41	C	11.47 ± 0.80	B	12.66 ± 0.68	C	0.89 ± 0.05	B	1.46 ± 0.03	D	26.48 ± 1.37	C						
50F + 75I + PGPR	651 ± 46	B	818 ± 41	B	9.40 ± 0.80	A	14.18 ± 0.68	C	0.85 ± 0.05	B	1.47 ± 0.03	D	25.89 ± 1.37	ABC						
**Fully ripe stage**																				
100F + 100I	1188 ± 27	D	1196 ± 28	D	7.60 ± 0.58	A	11.79 ± 0.54	A	1.75 ± 0.062	A	2.03 ± 0.04	B	23.16 ± 1.10	A	162 ± 5	B	51 ± 2	A	213 ± 8	BC
50F + 100I	1125 ± 27	CD	1131 ± 28	CD	9.74 ± 0.58	BC	12.71 ± 0.54	A	1.60 ± 0.062	A	1.93 ± 0.04	AB	25.98 ± 1.10	AB	164 ± 5	B	53 ± 2	A	217 ± 8	C
50F + 100I + PGPR	1089 ± 27	C	1099 ± 28	C	7.18 ± 0.58	A	11.41 ± 0.54	A	2.10 ± 0.062	B	1.85 ± 0.04	A	22.54 ± 1.10	A	145 ± 5	A	49 ± 2	A	194 ± 8	AB
100F + 75I	1059 ± 27	C	1066 ± 28	C	7.78 ± 0.58	A	12.28 ± 0.54	A	2.06 ± 0.062	B	2.28 ± 0.04	C	24.40 ± 1.10	A	137 ± 5	A	52 ± 2	A	189 ± 8	A
50F + 75I	898 ± 27	B	967 ± 28	B	9.41 ± 0.58	B	12.35 ± 0.54	A	1.77 ± 0.062	A	2.19 ± 0.04	C	25.73 ± 1.10	AB	137 ± 5	A	54 ± 2	A	191 ± 8	AB
50F + 75I + PGPR	652 ± 27	A	667 ± 28	A	11.27 ± 0.58	C	13.05 ± 0.54	A	1.99 ± 0.062	B	2.16 ± 0.04	C	28.47 ± 1.10	B	141 ± 5	A	47 ± 2	A	188 ± 8	A

Data are the mean of twenty-one replicates (LS Mean ± LS SE). Different uppercase letters indicate significant differences among treatments according to the Duncan test ($p < 0.05$).

Table S3. Mean content of ascorbic acid and vitamin C in green-ripe pepper fruits of the evaluated genotypes and treatments.

Genotype	100F+100I			50F+100I			50F+100I+PGPR			100F+75I			50F+75I			50F+75I+PGPR		
	**Ascorbic acid (mg kg^{-1})**																	
BGV13004	584 ± 186	a [1]	AB [2]	1116 ± 159	d	CD	398 ± 96	ab	A	1333 ± 30	e	D	905 ± 60	bc	BC	797 ± 110	bc	BC
Najerano	430 ± 80	a	AB	328 ± 68	a	A	387 ± 80	ab	A	849 ± 147	abc	B	407 ± 126	a	AB	335 ± 78	a	A
Piquillo	740 ± 111	ab	BC	668 ± 25	bc	BC	298 ± 152	a	A	782 ± 127	ab	C	706 ± 37	ab	BC	392 ± 41	a	AB
H1	623 ± 211	ab	A	1026 ± 55	d	B	651 ± 94	abc	A	1209 ± 72	de	B	1229 ± 149	c	B	1061 ± 113	c	B
H2	958 ± 165	ab	A	679 ± 124	bc	A	775 ± 103	bc	A	989 ± 100	bcd	A	861 ± 80	b	A	565 ± 137	ab	A
Isabel F1	1163 ± 92	b	C	843 ± 19	cd	AB	852 ± 191	c	AB	1150 ± 85	cde	BC	993 ± 186	bc	ABC	830 ± 102	bc	A
Cabañeros F1	560 ± 243	a	A	411 ± 131	ab	A	255 ± 110	a	A	541 ± 103	a	A	677 ± 64	ab	A	577 ± 161	ab	A
	**Vitamin C (mg kg^{-1})**																	
BGV13004	714 ± 194	ab	AB	1250 ± 146	e	CD	572 ± 98	abc	A	1442 ± 13	d	D	984 ± 77	b	BC	929 ± 88	b	ABC
Najerano	507 ± 66	a	A	461 ± 50	ab	A	514 ± 86	ab	A	907 ± 141	ab	B	638 ± 99	a	AB	572 ± 76	a	A
Piquillo	1010 ± 113	ab	A	925 ± 30	cd	A	714 ± 146	bc	A	980 ± 123	b	A	1022 ± 44	b	A	781 ± 33	ab	A
H1	747 ± 171	ab	A	1103 ± 75	de	BC	801 ± 74	bc	AB	1309 ± 73	cd	C	1296 ± 144	c	C	1215 ± 103	c	C
H2	968 ± 164	ab	A	733 ± 120	bc	A	878 ± 126	c	A	1044 ± 79	bc	A	915 ± 80	ab	A	701 ± 118	ab	A
Isabel F1	1168 ± 90	b	A	852 ± 26	cd	A	895 ± 116	c	A	1150 ± 85	bcd	A	993 ± 96	b	A	860 ± 74	ab	A
Cabañeros F1	621 ± 226	a	AB	435 ± 124	a	AB	317 ± 98	a	A	644 ± 88	a	AB	787 ± 32	ab	B	666 ± 109	ab	AB

Data are the mean of three replicates (Mean ± SE). [1] Different lowercase letters indicate significant differences among genotypes within a treatment; [2] different uppercase letters indicate significant differences among treatments within a genotype (Duncan's test, $p < 0.05$).

Table S4. Mean content of ascorbic acid and vitamin C in fully ripe pepper fruits of the evaluated genotypes and treatments.

Genotype	100F+100I			50F+100I			50F+100I+PGPR			100F+75I			50F+75I			50F+75I+PGPR		
	**Ascorbic acid (mg kg^{-1})**																	
BGV13004	1565 ± 77	c [1]	D [2]	1203 ± 141	bc	BC	1305 ± 99	c	C	949 ± 33	ab	AB	876 ± 36	b	A	772 ± 61	c	A
Najerano	1021 ± 6	a	ABC	995 ± 32	b	ABC	1039 ± 45	ab	BC	1122 ± 34	b	C	799 ± 78	ab	A	845 ± 137	c	AB
Piquillo	1222 ± 100	b	B	1240 ± 64	c	B	1107 ± 62	abc	AB	858 ± 23	a	A	947 ± 41	bc	AB	855 ± 150	c	A
H1	1177 ± 63	ab	BC	1318 ± 18	c	C	1091 ± 113	abc	BC	1056 ± 16	ab	B	971 ± 131	bc	B	635 ± 46	abc	A
H2	1065 ± 52	ab	B	1168 ± 65	bc	B	970 ± 65	ab	B	1070 ± 66	ab	B	644 ± 28	a	A	677 ± 126	bc	A
Isabel F1	1181 ± 55	ab	B	1188 ± 67	bc	B	1197 ± 48	bc	B	1467 ± 124	c	C	1143 ± 19	c	B	443 ± 34	ab	A
Cabañeros F1	1083 ± 11	ab	C	766 ± 21	a	B	912 ± 57	a	B	889 ± 82	a	B	904 ± 37	b	B	340 ± 16	a	A
	**Vitamin C (mg kg^{-1})**																	
BGV13004	1602 ± 61	d	C	1203 ± 141	bc	B	1317 ± 93	c	B	949 ± 33	ab	A	921 ± 38	b	A	800 ± 70	c	A
Najerano	1021 ± 6	a	AB	1003 ± 27	b	AB	1039 ± 45	ab	AB	1144 ± 43	b	B	1008 ± 55	bc	AB	871 ± 145	c	A
Piquillo	1243 ± 91	c	B	1261 ± 71	c	B	1115 ± 61	abc	AB	862 ± 20	a	A	1077 ± 25	bc	AB	893 ± 158	c	A
H1	1177 ± 63	abc	BC	1325 ± 25	c	C	1111 ± 126	abc	BC	1068 ± 7	ab	B	990 ± 133	bc	B	635 ± 46	abc	A
H2	1065 ± 52	ab	B	1173 ± 68	bc	B	972 ± 67	ab	B	1073 ± 66	ab	B	650 ± 25	a	A	678 ± 127	bc	A
Isabel F1	1181 ± 55	bc	B	1188 ± 67	bc	B	1197 ± 48	bc	B	1468 ± 124	c	C	1143 ± 19	c	B	443 ± 34	ab	A
Cabañeros F1	1083 ± 11	abc	D	766 ± 21	a	B	941 ± 33	a	C	897 ± 87	a	BC	978 ± 42	bc	CD	349 ± 21	a	A

Data are the mean of three replicates (Mean ± SE). [1] Different lowercase letters indicate significant differences among genotypes within a treatment; [2] different uppercase letters indicate significant differences among treatments within a genotype (Duncan's test, $p < 0.05$).

Table S5. Mean content of quercetin, luteolin, kaempferol, apigenin and total flavonoids in green-ripe pepper fruits of the evaluated genotypes and treatments.

Genotype	100F+100I			50F+100I			50F+100I+PGPR			100F+75I			50F+75I			50F+75I+PGPR		
**Quercetin (mg kg⁻¹)**																		
BGV13004	12.69 ± 6.24	a [1]	A [2]	10.39 ± 0.97	ab	A	9.85 ± 1.05	ab	A	6.11 ± 0.71	a	A	12.34 ± 2.72	ab	A	5.60 ± 0.66	ab	A
Najerano	9.45 ± 1.31	a	AB	9.79 ± 1.82	ab	AB	9.99 ± 0.61	ab	AB	11.54 ± 0.49	b	AB	14.53 ± 2.53	b	B	8.67 ± 0.56	bcd	A
Piquillo	11.52 ± 0.27	a	ABC	13.32 ± 1.11	b	BC	12.04 ± 2.57	ab	ABC	8.43 ± 0.84	ab	A	10.35 ± 1.36	ab	AB	15.83 ± 2.04	d	C
H1	9.63 ± 2.00	a	A	13.66 ± 4.23	b	A	12.64 ± 3.01	ab	A	9.00 ± 2.01	ab	A	8.39 ± 0.87	ab	A	9.57 ± 0.99	cd	A
H2	8.85 ± 0.74	a	B	16.06 ± 2.30	b	C	14.33 ± 1.11	b	C	8.29 ±0.60	ab	B	11.53 ± 1.42	ab	BC	5.30 ± 0.57	a	A
Isabel F1	7.64 ± 0.64	a	A	6.90 ± 0.39	a	A	7.93 ± 0.69	a	A	8.27 ± 0.95	ab	A	7.69 ± 0.94	a	A	8.55 ± 3.02	abc	A
Cabañeros F1	11.60 ± 5. 19	a	A	9.83 ± 1.89	ab	A	9.85 ± 1.57	ab	A	8.18 ± 1.44	ab	A	15.46 ± 2.55	b	A	12.29 ± 3.57	cd	A
**Luteolin (mg kg⁻¹)**																		
BGV13004	12.69 ± 4.38	bc	A	10.79 ± 1.08	b	A	11.54 ± 0.96	b	A	7.76 ± 1.83	ab	A	12.74 ± 2.29	ab	A	7.98 ± 0.99	ab	A
Najerano	5.82 ± 0.74	a	A	7.71 ± 0.85	a	AB	7.71 ± 1.16	a	AB	6.75 ± 0.24	a	AB	8.18 ± 1.22	a	AB	9.65 ± 0.25	bc	B
Piquillo	20.46 ± 2.19	c	A	21.09 ± 1.40	c	A	21.23 ± 2.94	c	A	16.60 ± 0.79	d	A	19.71 ± 4.27	c	A	38.59 ± 3.92	d	B
H1	8.76 ± 0.59	ab	A	9.08 ± 1.22	ab	A	11.55 ± 2.12	ab	A	9.82 ± 0.90	bc	A	11.81 ± 0.49	ab	A	10.64 ± 1.14	bc	A
H2	7.41 ± 0.24	ab	AB	11.12 ± 1.32	b	BC	9.91 ± 1.28	ab	ABC	6.92 ± 0.45	ab	A	12.42 ± 1.67	ab	C	6.76 ± 1.39	a	A
Isabel F1	8.36 ± 2.21	ab	A	7.39 ± 0.17	a	A	8.92 ± 0.13	ab	A	5.98 ± 0.67	a	A	8.30 ± 0.09	a	A	8.94 ± 2.12	ab	A
Cabañeros F1	12.22 ± 2.38	abc	A	11.47 ± 1.09	b	A	12.48 ± 1.25	b	A	13.49 ± 0.76	cd	A	15.50 ± 2.42	c	A	16.68 ± 3.28	cd	A
**Kaempferol (mg kg⁻¹)**																		
BGV13004	0.60 ± 0.23	a	AB	0.78 ± 0.12	a	AB	0.74 ± 0.05	ab	AB	0.72 ± 0.04	bc	AB	0.96 ± 0.11	a	B	0.53 ± 0.05	a	A
Najerano	0.57 ± 0.05	a	A	0.55 ± 0.11	a	A	0.55 ± 0.10	a	A	0.72 ± 0.05	bc	A	0.76 ± 0.11	a	A	0.68 ± 0.04	b	A
Piquillo	0.65 ± 0.03	a	B	0.58 ± 0.05	a	AB	0.62 ± 0.08	ab	AB	0.49 ± 0.02	a	A	0.73 ± 0.08	a	BC	1.06 ± 0.20	cd	C
H1	0.55 ± 0.06	a	A	0.66 ± 0.23	a	AB	0.71 ± 0.10	ab	AB	0.62 ± 0.01	abc	AB	0.98 ± 0.10	a	B	0.75 ± 0.08	bc	AB
H2	0.50 ± 0.03	a	A	0.75 ± 0.11	a	BC	0.91 ± 0.09	b	C	0.82 ± 0.06	c	BC	0.93 ± 0.11	a	C	0.60 ± 0.01	ab	AB
Isabel F1	0.55 ± 0.04	a	AB	0.40 ± 0.01	a	A	0.57 ± 0.07	a	AB	0.51 ± 0.07	ab	A	0.76 ± 0.08	a	BC	1.00 ± 0.14	cd	C
Cabañeros F1	1.07 ± 0.58	a	A	0.65 ± 0.07	a	A	0.72 ± 0.11	ab	A	0.70 ± 0.13	abc	A	1.12 ± 0.21	a	A	1.34 ± 0.25	d	A
**Apigenin (mg kg⁻¹)**																		
BGV13004	1.11 ± 0.04	b	A	1.17 ± 0.03	a	AB	1.25 ± 0.05	c	AB	1.27 ± 0.05	b	AB	1.30 ± 0.08	a	B	1.19 ± 0.05	a	AB
Najerano	0.99 ± 0.03	ab	A	1.05 ± 0.03	a	AB	1.08 ± 0.03	ab	BC	1.25 ± 0.04	b	D	1.19 ± 0.03	a	CD	1.17 ± 0.02	a	CD
Piquillo	1.71 ± 0.06	c	A	1.89 ± 0.04	b	A	1.75 ± 0.07	d	A	1.71 ± 0.06	c	A	2.12 ± 0.06	b	B	2.62 ± 0.07	b	C
H1	0.93 ± 0.03	a	A	1.16 ± 0.10	a	B	1.18 ± 0.05	bc	B	1.28 ± 0.01	b	BC	1.48 ± 0.08	a	C	1.39 ± 0.09	a	C
H2	1.02 ± 0.05	ab	A	1.07 ± 0.08	a	A	1.16 ± 0.04	bc	AB	1.13 ± 0.06	a	AB	1.65 ± 0.34	a	B	1.21 ± 0.09	a	AB
Isabel F1	1.13 ± 0.04	b	ABC	1.08 ± 0.04	a	AB	1.04 ± 0.04	a	A	1.04 ± 0.01	a	A	1.19 ± 0.04	a	BC	1.26 ± 0.07	a	C
Cabañeros F1	1.06 ± 0.10	ab	A	1.14 ± 0.08	a	AB	1.04 ± 0.03	a	A	1.36 ± 0.05	b	B	1.26 ± 0.08	a	AB	1.43 ± 0.14	a	B
**Total flavonoids (mg kg⁻¹)**																		
BGV13004	27.08 ± 10.82	ab	A	23.13 ± 2.16	bc	A	23.38 ± 1.49	abc	A	15.86 ± 2.55	a	A	27.35 ± 4.82	ab	A	15.30 ± 1.33	ab	A
Najerano	16.83 ± 2.04	a	A	19.09 ± 2.64	ab	A	19.33 ± 1.56	ab	A	20.26 ± 0.74	abc	A	24.65 ± 3.79	ab	A	20.17 ± 0.85	bc	A
Piquillo	34.34 ± 2.35	b	A	36.88 ± 2.50	c	A	35.64 ± 5.64	d	A	27.23 ± 1.70	c	A	32.91 ± 5.54	b	A	58.10 ± 2.71	c	B
H1	19.87 ± 2.65	ab	A	24.55 ± 5.64	bc	A	26.07 ± 4.73	bcd	A	20.72 ± 2.40	abc	A	22.66 ± 1.16	ab	A	22.34 ± 2.18	c	A
H2	17.78 ± 1.05	ab	B	29.00 ± 3.51	c	C	26.31 ± 2.45	cd	C	17.14 ± 0.33	ab	B	26.54 ± 3.44	ab	C	13.86 ± 1.52	a	A
Isabel F1	17.68 ± 2.76	ab	A	15.77 ± 0.54	a	A	18.46 ± 0.66	a	A	15.80 ± 1.68	a	A	17.93 ± 1.04	a	A	19.75 ± 3.12	bc	A
Cabañeros F1	25.95 ± 7.66	ab	A	23.09 ± 2.02	bc	A	24.08 ± 1.76	abcd	A	23.72 ± 2.22	bc	A	33.30 ± 5.24	b	A	31.74 ± 7.21	cd	A

Data are the mean of three replicates (Mean ± SE). [1] Different lowercase letters indicate significant differences among genotypes within a treatment; [2] different uppercase letters indicate significant differences among treatments within a genotype (Duncan's test, $p < 0.05$).

Table S6. Mean content of quercetin, luteolin, kaempferol, apigenin and total flavonoids in fully ripe pepper fruits of the evaluated genotypes and treatments.

Genotype	100F+100I			50F+100I			50F+100I+PGPR			100F+75I			50F+75I			50F+75I+PGPR		
**Quercetin (mg kg⁻¹)**																		
BGV13004	6.80 ± 1.01	a [1]	A [2]	9.09 ± 11.90	a	A	5.88 ± 0.77	a	A	7.46 ± 0.50	ab	A	10.19 ± 3.72	ab	A	10.01 ± 0.83	a	A
Najerano	5.85 ± 0.20	a	A	10.95 ± 3.21	a	AB	8.79 ± 0.70	a	AB	10.93 ± 2.02	b	AB	7.47 ± 0.58	ab	AB	13.34 ± 2.86	a	B
Piquillo	11.45 ± 1.08	b	B	11.05 ± 1.48	a	B	6.05 ± 0.90	a	A	6.56 ± 0.59	a	A	12.24 ± 1.50	ab	B	13.01 ± 1.38	a	B
H1	7.75 ± 1.80	ab	A	8.59 ± 1.72	a	A	6.58 ± 1.13	a	A	8.43 ± 1.27	ab	A	8.62 ± 0.55	ab	A	9.95 ± 1.35	a	A
H2	7.31 ± 0.32	ab	A	12.98 ± 2.22	a	B	7.35 ± 0.89	a	A	8.06 ± 0.72	ab	AB	13.25 ± 3.65	b	AB	11.29 ± 2.03	a	AB
Isabel F1	7.80 ± 1.18	ab	A	7.29 ± 0.95	a	A	6.47 ± 1.12	a	A	6.38 ± 0.23	a	A	5.85 ± 0.26	a	A	13.36 ± 1.10	a	B
Cabañeros F1	6.22 ± 0.22	a	A	8.23 ± 0.53	a	A	9.11 ± 1.97	a	A	6.62 ± 0.62	a	A	8.25 ± 1.39	ab	A	7.96 ± 1.99	a	A
**Luteolin (mg kg⁻¹)**																		
BGV13004	9.53 ± 0.09	ab	A	11.90 ± 0.74	a	A	10.56 ± 0.57	abc	A	12.12 ± 0.77	b	A	12.44 ± 2.97	bc	A	13.25 ± 1.12	a	A
Najerano	9.08 ± 0.66	a	A	9.63 ± 1.42	a	A	9.58 ± 0.13	ab	A	7.83 ± 1.61	a	A	6.96 ± 0.44	a	A	8.98 ± 1.18	a	A
Piquillo	22.10 ± 1.97	c	AB	22.05 ± 3.02	b	AB	14.91 ± 2.15	d	A	21.12 ± 3.64	c	AB	21.69 ± 1.83	d	A	24.03 ± 2.37	b	B
H1	11.72 ± 0.59	b	A	12.40 ± 1.63	a	A	11.94 ± 0.35	bcd	A	12.81 ± 1.33	b	A	11.48 ± 0.84	bc	A	13.25 ± 2.08	a	A
H2	10.31 ± 0.76	ab	A	10.00 ± 1.18	a	A	8.84 ± 0.27	a	A	10.25 ± 0.61	ab	A	9.83 ± 1.46	b	A	10.50 ± 1.44	a	A
Isabel F1	10.18 ± 1.12	ab	A	10.52 ± 0.72	a	A	10.36 ± 0.26	abc	A	9.96 ± 0.39	ab	A	9.73 ± 0.33	bc	A	11.12 ± 0.63	a	A
Cabañeros F1	9.60 ± 0.37	ab	A	12.45 ± 0.73	a	AB	13.66 ± 2.30	cd	AB	11.87 ± 0.90	b	AB	14.33 ± 1.61	cd	B	10.22 ± 0.92	a	AB
**Kaempferol (mg kg⁻¹)**																		
BGV13004	1.81 ± 0.11	bc	AB	1.52 ± 0.07	ab	A	2.10 ± 0.18	ab	ABC	2.46 ± 0.04	b	C	2.19 ± 0.35	a	BC	1.82 ± 0.09	a	AB
Najerano	1.63 ± 0.08	ab	A	1.55 ± 0.23	ab	A	2.43 ± 0.12	b	B	2.27 ± 0.16	b	B	1.69 ± 0.17	a	A	2.39 ± 0.12	b	B
Piquillo	2.08 ± 0.08	c	A	2.00 ± 0.22	b	A	1.79 ± 0.25	a	A	1.61 ± 0.07	a	A	1.70 ± 0.21	a	A	2.11 ± 0.03	ab	A
H1	2.13 ±0.22	c	AB	1.79 ± 0.10	ab	A	2.12 ± 0.14	ab	AB	2.35 ± 0.16	b	B	1.68 ± 0.16	a	A	1.90 ± 0.12	a	AB
H2	2.05 ± 0.19	bc	A	1.67 ± 0.22	ab	A	2.04 ± 0.02	ab	A	2.04 ± 0.21	ab	A	1.54 ± 0.33	a	A	2.00 ± 0.02	a	A
Isabel F1	1.20 ± 0.10	a	A	1.33 ± 0.04	a	A	1.94 ± 0.02	ab	C	2.01 ± 0.07	ab	C	1.58 ± 0.04	a	B	1.95 ± 0.04	a	C
Cabañeros F1	1.33 ± 0.13	a	A	1.32 ± 0.08	a	A	2.30 ± 0.26	ab	B	1.70 ± 0.14	a	AB	2.04 ± 0.27	a	B	1.73 ± 0.25	a	AB
**Apigenin (mg kg⁻¹)**																		
BGV13004	1.95 ± 0.10	b	A	1.76 ± 0.16	a	A	1.78 ± 0.05	a	A	2.27 ± 0.02	c	B	2.04 ± 0.08	ab	AB	1.97 ± 0.03	a	A
Najerano	1.93 ± 0.08	b	C	1.71 ± 0.10	a	AB	1.65 ± 0.05	a	A	1.94 ± 0.03	b	C	1.84 ± 0.04	a	ABC	1.89 ± 0.04	a	BC
Piquillo	3.22 ± 0.12	c	ABC	2.97 ± 0.23	b	AB	2.60 ± 0.13	b	A	3.76 ± 0.35	d	C	3.24 ± 0.12	c	BC	3.34 ± 0.09	b	BC
H1	1.75 ± 0.11	ab	A	1.96 ± 0.03	a	ABC	1.80 ± 0.06	a	AB	2.16 ± 0.03	c	C	2.20 ± 0.09	b	C	2.01 ± 0.11	a	BC
H2	1.68 ± 0.03	ab	A	1.66 ± 0.06	a	A	1.71 ± 0.03	a	A	2.19 ± 0.04	c	B	1.88 ± 0.16	a	AB	1.93 ± 0.13	a	AB
Isabel F1	2.05 ± 0.23	b	B	1.74 ± 0.11	a	AB	1.65 ± 0.02	a	A	1.98 ± 0.04	b	AB	2.10 ± 0.05	ab	B	2.04 ± 0.04	a	B
Cabañeros F1	1.60 ± 0.04	a	A	1.72 ± 0.09	a	AB	1.76 ± 0.10	a	AB	1.68 ± 0.05	a	A	2.05 ± 0.12	ab	C	1.95 ± 0.03	a	BC
**Total flavonoids (mg kg⁻¹)**																		
BGV13004	20.10 ± 1.04	a	A	24.27 ± 0.65	a	A	20.32 ± 1.51	a	A	24.30 ± 1.30	ab	A	26.86 ± 7.11	abc	A	27.04 ± 1.15	ab	A
Najerano	18.48 ± 0.63	a	A	23.85 ± 4.94	a	A	22.45 ± 0.87	a	A	22.97 ± 3.48	a	A	17.96 ± 1.15	a	A	26.60 ± 3.96	a	A
Piquillo	38.86 ± 3.07	b	B	38.07 ± 4.78	b	B	25.34 ± 3.06	a	A	33.06 ± 4.62	b	AB	38.88 ± 3.45	c	B	42.49 ± 3.84	b	B
H1	23.35 ± 2.21	a	A	24.74 ± 3.32	a	A	22.44 ± 1.18	a	A	25.75 ± 2.77	ab	A	23.97 ± 1.32	abc	A	27.11 ± 2.77	ab	A
H2	21.36 ± 1.10	a	A	26.31 ± 3.67	ab	A	19.94 ± 0.95	a	A	22.54 ± 1.30	ab	A	26.50 ± 5.53	abc	A	25.72 ± 3.58	a	A
Isabel F1	21.22 ± 2.51	a	A	20.88 ± 1.67	a	A	20.42 ± 1.16	a	A	20.33 ± 0.72	a	A	19.26 ± 0.56	ab	A	28.48 ± 1.60	ab	B
Cabañeros F1	18.75 ± 0.25	a	A	23.73 ± 1.20	a	A	26.83 ± 4.60	a	A	21.87 ± 1.41	a	A	26.67 ± 3.15	bc	A	21.86 ± 2.84	a	A

Data are the mean of three replicates (Mean ± SE). [1] Different lowercase letters indicate significant differences among genotypes within a treatment; [2] different uppercase letters indicate significant differences among treatments within a genotype (Duncan's test, $p < 0.05$).

Table S7. Mean content of red, yellow-orange and total carotenoids in fully ripe pepper fruits of the evaluated genotypes and treatments.

	**Red carotenoids (mg kg⁻¹)**																	
**Genotype**	**100F+100I**			**50F+100I**			**50F+100I+PGPR**			**100F+75I**			**50F+75I**			**50F+75I+PGPR**		
BGV13004	219 ± 17	c [1]	C [2]	178 ± 20	cd	BC	165 ± 12	c	AB	126 ± 7	ab	A	155 ± 9	bcd	AB	152 ± 16	bc	AB
Najerano	147 ± 20	b	AB	122 ± 17	ab	A	150 ± 3	bc	AB	166 ± 9	ab	B	130 ± 15	bc	AB	155 ± 4	bc	AB
Piquillo	222 ± 7	c	C	221 ± 6	de	C	153 ± 3	bc	A	167 ± 10	b	AB	195 ± 11	d	BC	191 ± 14	c	B
H1	164 ± 5	b	A	234 ± 24	e	B	173 ± 4	c	A	151 ± 25	ab	A	168 ± 19	cd	A	132 ± 8	ab	A
H2	170 ± 4	b	A	174 ± 7	cd	A	131 ± 2	ab	A	135 ± 40	ab	A	115 ± 17	ab	A	147 ± 6	b	A
Isabel F1	106 ± 10	a	AB	141 ± 19	bc	B	126 ± 1	ab	AB	118 ± 7	ab	AB	122 ± 16	abc	AB	92 ± 2	a	A
Cabañeros F1	107 ± 10	a	A	79 ± 8	a	A	120 ± 17	a	A	98 ± 20	a	A	77 ± 14	a	A	119 ± 24	ab	A
	**Yellow – Orange carotenoids (mg kg⁻¹)**																	
BGV13004	65 ± 5	d	B	48 ± 15	abc	AB	47 ± 4	ab	AB	33 ± 1	a	A	54 ± 4	bc	AB	42 ± 5	bc	AB
Najerano	43 ± 8	bc	A	39 ± 5	ab	A	44 ± 2	ab	A	72 ± 4	b	B	49 ± 5	abc	A	53 ± 3	c	A
Piquillo	87 ± 9	e	AB	97 ± 12	d	B	67 ± 7	d	A	70 ± 1	b	A	96 ± 9	d	B	82 ± 4	d	AB
H1	52 ± 5	cd	AB	74 ± 6	cd	B	61 ± 3	cd	AB	57 ± 10	ab	AB	64 ± 9	c	AB	42 ± 3	bc	A
H2	56 ± 2	cd	A	53 ± 3	bc	A	50 ± 2	bc	A	57 ± 16	ab	A	46 ± 6	abc	A	52 ± 1	c	A
Isabel F1	29 ± 5	ab	AB	41 ± 8	ab	B	34 ± 1	a	AB	40 ± 2	a	B	37 ± 5	ab	AB	23 ± 0	a	A
Cabañeros F1	23 ± 2	a	A	22 ± 3	a	A	36 ± 6	a	A	35 ± 5	a	A	28 ± 7	a	A	35 ± 6	b	A
	**Total carotenoids (mg kg⁻¹)**																	
BGV13004	284 ± 21	c	B	225 ± 34	b	AB	212 ± 16	bc	A	159 ± 8	ab	A	209 ± 14	bc	A	194 ± 20	b	A
Najerano	190 ± 27	b	AB	161 ± 21	ab	A	193 ± 5	ab	AB	238 ± 13	b	B	179 ± 19	bc	A	207 ± 6	b	AB
Piquillo	309 ± 14	c	C	318 ± 18	c	C	220 ± 10	bc	A	237 ± 10	b	AB	291 ± 20	d	C	274 ± 18	c	BC
H1	216 ± 9	b	A	308 ± 30	c	B	234 ± 6	c	A	208 ± 35	ab	A	232 ± 28	cd	A	174 ± 11	b	A
H2	226 ± 5	b	A	227 ± 10	b	A	182 ± 3	ab	A	192 ± 56	ab	A	161 ± 23	ab	A	199 ± 5	b	A
Isabel F1	134 ± 14	a	AB	182 ± 26	b	B	160 ± 1	a	AB	157 ± 8	ab	AB	160 ± 22	ab	AB	116 ± 2	a	A
Cabañeros F1	130 ± 12	a	A	100 ± 11	a	A	156 ± 23	a	A	133 ±25	a	A	105 ± 20	a	A	155 ± 30	ab	A

Data are the mean of three replicates (Mean ± SE). [1] Different lowercase letters indicate significant differences among genotypes within a treatment; [2] different uppercase letters indicate significant differences among treatments within a genotype (Duncan's test, $p < 0.05$).

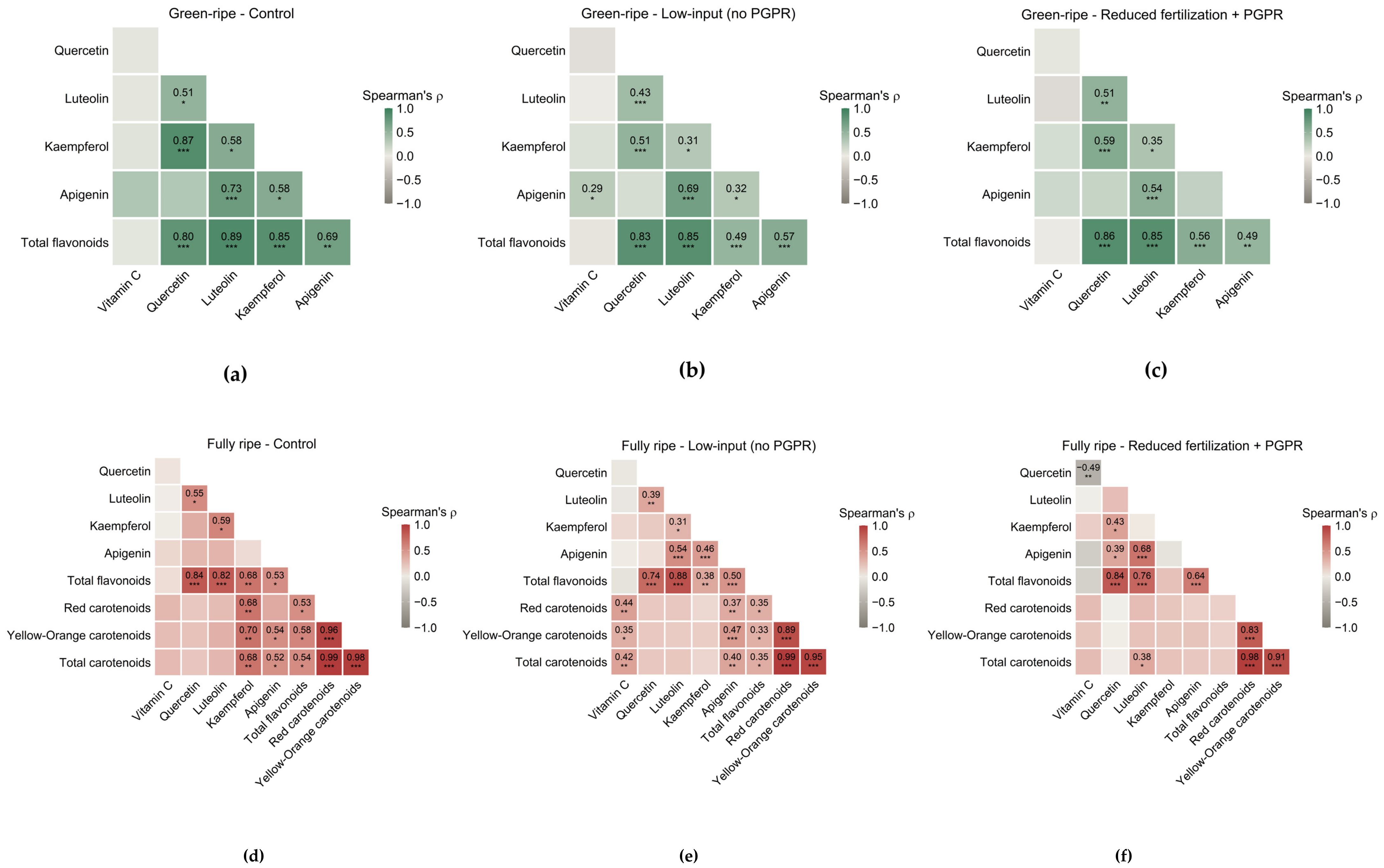
Green-ripe - Control
Quercetin
Luteolin
Kaempferol
Apigenin
Total flavonoids
Vitamin C
Spearman's ρ
1.0
0.5
0.0
−0.5
−1.0
0.51 *
0.87 ***
0.58 *
0.73 ***
0.58 *
0.80 ***
0.89 ***
0.85 ***
0.69 **
(a)
Green-ripe - Low-input (no PGPR)
0.43 ***
0.51 ***
0.31 *
0.29 *
0.69 ***
0.32 *
0.83 ***
0.85 ***
0.49 ***
0.57 ***
(b)
Green-ripe - Reduced fertilization + PGPR
0.51 **
0.59 ***
0.35 *
0.54 ***
0.86 ***
0.85 ***
0.56 ***
0.49 **
(c)
Fully ripe - Control
Red carotenoids
Yellow-Orange carotenoids
Total carotenoids
0.55 *
0.59 *
0.84 ***
0.82 ***
0.68 **
0.53 *
0.68 **
0.53 *
0.70 **
0.54 *
0.58 *
0.96 ***
0.68 **
0.52 *
0.54 *
0.99 ***
0.98 ***
(d)
Fully ripe - Low-input (no PGPR)
0.39 **
0.31 *
0.54 ***
0.46 ***
0.74 ***
0.88 ***
0.38 **
0.50 ***
0.44 **
0.37 **
0.35 *
0.35 *
0.47 ***
0.33 *
0.89 ***
0.42 **
0.40 **
0.35 *
0.99 ***
0.95 ***
(e)
Fully ripe - Reduced fertilization + PGPR
−0.49 **
0.43 *
0.39 *
0.68 ***
0.84 ***
0.76 ***
0.64 ***
0.83 ***
0.38 *
0.98 ***
0.91 ***
(f)

Figure S1. Spearman's rank correlation (ρ) heatmap for green-ripe stage: **(a)** Control, **(b)** Low-input without PGPR, and **(c)** Reduced fertilization with PGPR categories; and for the fully ripe stage: **(d)** Control, **(e)** Low-input without PGPR, and **(f)** Reduced fertilization with PGPR categories. *, ** and *** indicate $p < 0.05$, 0.01 and 0.001, respectively.
